# Photochemical N‐dealkylation of Tertiary Amines Coupled With Photocharging of Poly(Heptazine Imides)

**DOI:** 10.1002/anie.202522677

**Published:** 2026-02-04

**Authors:** Jingru Zhuang, Quanhao Zhang, Chong Wang, Tao Yao, Cheuk Lok Cheng, Pavlo O. Dral, Oleksandr Savateev

**Affiliations:** ^1^ Department of Chemistry The Chinese University of Hong Kong Shatin Hong Kong China; ^2^ State Key Laboratory of Physical Chemistry of Solid Surfaces Department of Chemistry and Chemical Engineering College of Chemistry and Chemical Engineering and Fujian Provincial Key Laboratory of Theoretical and Computational Chemistry Xiamen University Xiamen Fujian China; ^3^ Institute of Physics Faculty of Physics Astronomy, and Informatics Nicolaus Copernicus University Toruń Poland; ^4^ Aitomistic Shenzhen China

**Keywords:** carbon nitride, dealkylation, photocharging, proton‐coupled electron transfer

## Abstract

Photocharging in carbon nitride materials is emerging as a promising route for light‐driven energy storage, yet the nature of the storage sites and their mechanisms remain elusive. Here, we investigate photocharging in poly(heptazine imide) (PHI), focusing on its protonated (H‐PHI) and sodium (Na‐PHI) forms. Using the photochemical dealkylation of triethylamine, we quantify the density of electron–proton (e^−^/H^+^) pairs stored in these materials and define a descriptor, *N*_e_:*N*_hept_, to track photocharging at the molecular level. H‐PHI demonstrates superior electron storage and reactivity, attributed to its microporous 2D structure and favorable energetics. Theoretical modeling, combined with spectroscopic analysis, reveals the nature of the active heptazine sites and their transformation during charging. The material retains its structural and functional integrity across multiple cycles, underscoring its stability and recyclability. These insights open new avenues for the rational design of carbon nitride‐based semiconductors for light‐induced redox applications.

## Graphical abstract

Protonated poly(heptazine imide) (H‐PHI) functions as an energy reservoir, thereby facilitating the endothermic dealkylation of triethylamine through a proton‐coupled two‐electron transfer (PC2ET) process. The extent of photocharging is quantitatively described by a molecular‐level descriptor, *N*_e_:*N*_hept_, providing a direct correlation between stored charge density and reactivity.

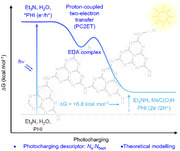

## 1 Introduction

**FIGURE 1:**
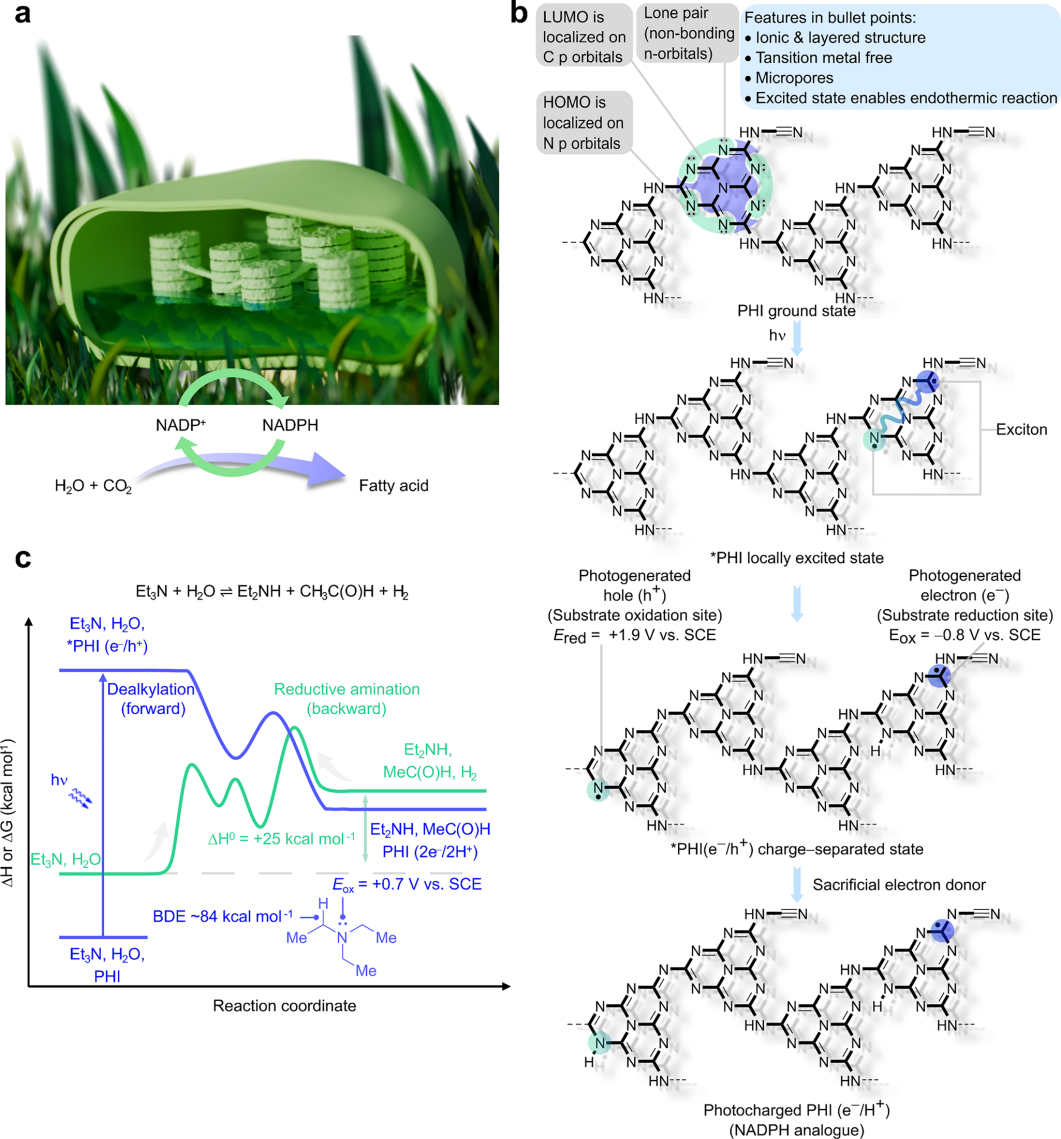
Photocharging of carbon nitride by means of dealkylation of tertiary amines, a process inspired by natural photosynthesis. (a) Synthesis of fatty acid by using CO_2_ and water in the chloroplast by photocharging NADP^+^ into NADPH. (b) Schematic illustration of HOMO and LUMO of PHI in the ground state; locally excited state; redox sites of PHI charge‐separated state; formation of photocharged PHI(e^−^/H^+^) in the presence of sacrificial electron donor. (c) A scheme of triethylamine dealkylation reaction profile executed thermally and photochemically using PHI as acceptor of electrons and protons, and the backward reductive amination reaction.

Growing global demand for sustainable energy has intensified the search for advanced energy storage materials that are efficient, economical, and environmentally friendly [1, 2]. Traditional energy storage systems often face challenges related to energy density, stability, and cost, highlighting the need for innovative materials [3]. Nature provides valuable inspiration for overcoming these challenges, particularly through photosynthesis—a highly efficient process for solar energy conversion and storage. A key component of this process is nicotinamide adenine dinucleotide phosphate (NADP^+^), which captures and stores solar energy in the form of NADPH [4]. In chloroplasts, NADPH acts as an electron donor, enabling chemical reactions such as fatty acid synthesis while continuously cycling back to NADP^+^ (Figure 1a) [5]. Inspired by this natural blueprint, considerable efforts have been devoted to the development of functional materials capable of absorbing photons in the near UV–visible range of the electromagnetic spectrum, followed by charge separation, and driving the redox reactions to form NADPH analogs [6]. These advancements hold promise for the next‐generation renewable energy storage solutions, bridging the gap between biological energy conversion and artificial systems [7, 8].

Graphitic carbon nitrides (g‐CNs), and, in particular, poly(heptazine imide) (PHI), have emerged as promising candidates. Their inherent ionic structure not only stabilizes the photogenerated electrons but also promotes effective photocharging—a feature central to achieving efficient solar energy storage [6, 9]. Recent conceptual breakthroughs, including “Dark Photocatalysis” and “Illumination‐Driven Electron Accumulation in Semiconductors and Exploitation (IDEASE),” have advanced our understanding of the fundamental processes governing carbon nitride photocharging [10, 11]. These concepts share a common mechanistic framework: upon light absorption, formation of an exciton and separation of charges occur, with the hole localized at nitrogen atoms of the heptazine units, while electrons remain associated with carbon atoms (see Supplementary Discussion 1 for more details). To achieve free electrons and holes, PHI in the locally excited state has to overcome the strong Coulomb interactions, namely the exciton binding energy (*E*_b_), to form the charge‐separate state. The ability of carbon nitride to undergo photocharging has been identified as a solution to reducing the exciton binding energy. This solution has been demonstrated to reduce the exciton binding energy, while also inhibiting photogenerated electron‐hole recombination [12].

When a sacrificial agent is introduced, the photogenerated holes are rapidly consumed, leading to the accumulation of electrons and protons (or other counter ions)—a process that is key to the material photocharging (Figure 1b) [6]. It can be exploited by coupling photocharging of g‐CN with a synthetically useful chemical reaction, such as selective dealkylation of amines, which serve as sacrificial donors of electrons (and protons) [13]—analogously to NADP^+^ reduction to NADPH with water as a “sacrificial agent” in biological systems. Using enthalpies of formation of triethylamine, water, diethylamine, acetaldehyde, and hydrogen in the gas phase [14, 15], our calculations indicate that dealkylation of tertiary amines is an endothermic reaction at 298 K. Under standard conditions, the equilibrium is shifted to reactants. The reversed reaction is known as reductive amination, which proceeds at elevated temperatures over transition metal catalysts [16, 17]. Therefore, photochemistry is one of a few approaches that may be used to execute dealkylation of tertiary amines without the need to add more complex reactants (Figure 1c) [18, 19, 20, 21]. Tertiary amines have relatively low oxidation potential (+0.7 V vs. SCE) [22], while the bond dissociation energy (BDE) of C−H next to the tertiary nitrogen atom is ∼84 kcal mol^−1^ [23]. Given the PHI electronically‐excited state reduction potential of +1.9 V versus SCE and the fact that it can cleave X−H bonds with BDE<117 kcal mol^−1^ [24], one‐electron oxidation of a tertiary amine and abstraction of a hydrogen atom from a tertiary amine are both thermodynamically feasible. We hypothesize that dealkylation of tertiary amines may be enabled photochemically by employing g‐CN. The key intermediate is the iminium cation, which upon hydrolysis delivers a secondary amine and aldehyde [22].

Despite promising developments in the area of carbon nitride photocharging, several critical challenges remain unresolved [25]. In particular, the effect of exciton binding energy on the photocharging reaction, the time factor on the rate of photocharging, and the density of stored electrons have not been studied in greater depth. From a theoretical perspective, the correlation between the Gibbs free energy change of a tertiary amine dealkylation reaction and the density of electrons stored in carbon nitride was not investigated. Conventional metrics, such as surface area, overall yield, or the total stored charge per gram, may not accurately reflect the contributions of individual heptazine units to electron/proton storage. Consequently, these metrics may not facilitate valid comparisons across diverse carbon‐nitride architectures (H‐PHI, Na‐PHI, mpg‐CN, g‐CN). From a fundamental perspective, an open question remains as to whether heptazine units behave as independent photoredox sites or whether photocharging of one heptazine unit affects the properties of others.

In this work, we present a comprehensive study of electron storage in PHI and coupling photocharging with the dealkylation of tertiary aliphatic amines. We elucidate the intricate relationship between the density of electrons stored in PHI and the Gibbs free energy change of the reaction. The results not only provide critical insights into the storage sites of electron/proton (e^−^/H^+^) couples but also highlight how gradual variations in molecular structure can dictate energy transfer and storage efficiency. By establishing a clear mechanistic understanding of electron storage in carbon nitride, this work provides an underlying logic for a deeper understanding of other materials capable of undergoing photocharging. In essence, researchers can utilize the structural units of a material as the foundation for a bottom‐up approach to identify and leverage the material's inherent photocharging capabilities.

## 2 Results and Discussion

**FIGURE 2:**
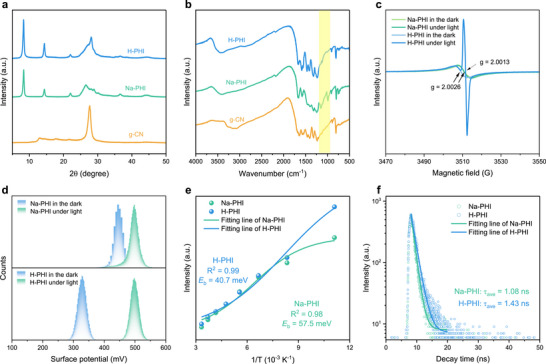
Characterization of the synthesized carbon nitrides. (a) PXRD patterns. (b) FT‐IR spectra. (c) EPR spectra of Na‐PHI and H‐PHI. Conditions: samples were recorded on CW EPR (X band) under central frequency (9.83 GHz), and attenuation (23 dB), 298 K. A 300 W Xe lamp with a 410 nm band pass filter was used to excite the sample. (d) KPFM measurements of the samples in the dark and upon light irradiation (410 nm, 27.5 ± 3 mW cm^−2^). (e) The PL intensity of Na‐PHI and H‐PHI as a function of the reciprocal of the operation temperature. (f) Time‐resolved PL decay of Na‐PHI and H‐PHI.

We synthesized sodium poly(heptazine imide) (Na‐PHI), protonated poly(heptazine imide) (H‐PHI), and melon‐type graphitic carbon nitride (g‐CN) and subjected them to a series of analyses. Overall, spectroscopic characterization of the materials unambiguously confirmed their structure. Below, we only focus on the most important aspects. Powder x‐ray diffraction (PXRD) analysis revealed higher crystallinity of PHI compared to melon‐type g‐CN, as evidenced by the presence of more intense reflections (Figure 2a). Treatment of Na‐PHI with acid to remove sodium ions did not alter the diffraction pattern, but increased the intensity of peaks, indicating that the layered structure was preserved and that H‐PHI exhibits improved structural order relative to Na‐PHI [26]. Conversely, melon‐type g‐CN exhibits broader and less defined features, reflecting its lower structural order [27, 28]. Scanning electron microscopy (SEM) revealed a similar morphology of all the samples—lamellar stacks (Figure S1). Compared to g‐CN and Na‐PHI, the high‐resolution transmission electron microscopy (HR‐TEM) images (Figure S2) of H‐PHI display crystalline regions with a lattice spacing calculated according to the Inverse Fast‐Fourier transform (IFFT, Figure S3) that coincides with that observed in PXRD patterns (Table S1). Fourier‐transform infrared (FT‐IR) spectra confirmed the presence of cyano‐groups (peak at 2180 cm^−1^) in both PHIs, which enhances their electron storage capacity [12]. In Na‐PHI, the peaks at 1155 and 988 cm^−1^ are assigned to vibrations of the metal‐CN_2_ groups. These peaks are absent in the FT‐IR spectrum of H‐PHI, corroborating the exchange of Na^+^ by H^+^ (highlighted in Figure 2b) [29]. X‐ray photoelectron spectroscopy (XPS) further confirmed the chemical state of constituting elements, C, N, Na, and Cl (Figure S4). The results confirmed the successful synthesis of g‐CN and Na‐PHI, and the effective removal of Na^+^ from Na‐PHI upon washing with acid and water [30]. XPS analysis for Cl element indicated that washing of H‐PHI with water was sufficient to remove Cl anions and, as such, eliminate all the adsorbed HCl. Specific surface areas of g‐CN (17 m^2^ g^−1^), Na‐PHI (24 m^2^ g^−1^), and H‐PHI (31 m^2^ g^−1^) were determined from N_2_ physisorption isotherms (Table S2 and Figure S5). Compared to melon‐type g‐CN, both Na‐PHI and H‐PHI display enhanced light absorption, with Na‐PHI exhibiting a red‐shifted edge and a weak absorption band associated with n‐π* electronic transition (Figure S6a) [31]. Overall, treatment of Na‐PHI with HCl widens the optical band gap of the material, which is consistent with a number of earlier reports [9, 26, 32, 33, 34, 35]. Combining data derived from the Tauc plots (Figure S6b) and the Mott–Schottky measurements (Figure S7), potentials of HOMO and LUMO in the samples were assigned (Figure S8) [36].

As demonstrated by photoelectrochemical measurements, treatment of Na‐PHI with HCl gives H‐PHI, which exhibits higher photocurrent density and lower charge‐transfer resistance compared to the pristine material (Figure S9). These observations are consistent with one report [33], but are different from another [37], which we explain by minute differences in sample preparation and electrode fabrication. Meanwhile, PHIs exhibited reduced photoluminescence (PL) intensity compared to melon‐type g‐CN, indicating suppressed radiative charge recombination (Figure S10) [38]. Compared to Na‐PHI, H‐PHI shows stronger PL intensity, which is consistent with other reports [26, 33, 37, 39]. Electron paramagnetic resonance (EPR) spectra recorded in the dark revealed resonance signals at *g* = 2.0026 (3505 G for H‐PHI; 3507 G for Na‐PHI), ascribed to unpaired electrons localized on carbon atoms of the heptazine units [40, 41]. In situ irradiation of H‐PHI at 410 nm gives an additional narrower signal at 3511 G with a *g* value of 2.0013 in the EPR spectrum. We attribute this signal to the photogenerated electron in the electronically excited state of H‐PHI (Figure 2c). Under light irradiation, more unpaired electrons are generated in H‐PHI and gradually accumulate with time (Figure S11; see Supplementary Discussion 5 for more details on the operation) [42]. Cessation of H‐PHI irradiation at 410 nm leads to the immediate disappearance of this narrow signal. Irradiation of Na‐PHI does not give the analogous signal in the EPR spectrum. To gain further insights into the photoexcitation properties of PHIs, Kelvin Probe Force Microscopy (KPFM) was employed (Figure 2d). H‐PHI exhibits a pronounced surface potential shift of 170.0 mV under light irradiation, compared to 45.7 mV for Na‐PHI (Figure S12 and Table S3). Temperature‐dependent PL spectra ranging from 90 to 300 K (Figure S13) were used to estimate the exciton binding energy (*E*_b_), yielding values of 57.5 meV for Na‐PHI and 40.7 meV for H‐PHI, based on data fitting using the equation in Supplementary Method 1.5. The magnitude of *E*_b_ values, a few tens of meV, is in the same range that was reported earlier for heptazine‐based carbon nitrides derived from similar temperature‐dependent PL measurements (Figure 2e) [43, 44]. The reduced *E*_b_ in H‐PHI suggests more efficient exciton dissociation [45], which is in agreement with its EPR behavior, and highlights its superior potential in photochemical applications. On the other hand, the H‐PHI electronically excited state possesses a slightly longer lifetime compared to Na‐PHI as inferred from the time‐resolved PL data (Figure 2f), which is consistent with previous reports [33, 37].

Having confirmed the structure and established the electronic features of the materials, we next examined their photocharging behavior coupled with the dealkylation of triethylamine (TEA). Unlike small organic sensitizers, which in solutions exist as quite independent photoredox species, heptazine units in PHIs are linked covalently. This implies that transfer and storage of an electron‐proton pair from a suitable hydrogen atom donor, such as TEA, to the electronically excited state of a small organic sensitizer molecule should not affect the energetics of the same reaction of other molecular sensitizers molecules. In the case of PHIs, the photoredox reaction at one heptazine unit can affect the photoredox properties of other heptazines. As a result, this may lead to a scenario in which the enthalpy change (Δ*H*) of the TEA photochemical dehydrogenation reaction depends on the density of electron‐proton pairs stored in the material. To quantify the extent of PHI photocharging, we introduce a parameter “*N*_e_:*N*_hept_”, as the ratio of electrons stored in PHI to the total number of heptazine units in the sample. For instance, a 1:1 ratio corresponds to the photocharged PHI, in which statistically one electron (and a proton) is stored per one heptazine unit, 1:2–one electron per two heptazine units, and so on. Based on the spectroscopic data and theoretical studies of triaryl heptazine—a molecular model of graphitic carbon nitride, the formation of heptazinyl radical was concluded earlier [46, 47]. In such a structure, one hydrogen atom (one electron‐proton pair) is added to the heptazine unit. While it is theoretically possible for each unit to store more than one electron and proton (*N*_e_:*N*_hept_ = n:m, where n > m), this remains unsupported by experimental evidence.

The results of solvent screening and other parameters are collected in Table S4. Acetonitrile was selected as the optimal polar solvent due to its redox stability and ability to stabilize PHI dispersions (Table 1, entry 1). Under the standard reaction conditions, *N*_e_:*N*_hept_ values for Na‐PHI and H‐PHI are approximately 1:4, which were derived from the yield of Et_2_NH, 24 and 27% respectively, and the molar weights of the PHIs' unit cells. These *N*_e_:*N*_hept_ ratios of 1:4 match those deduced from potassium poly(heptazine imide) (K‐PHI) photocharging using a solution of benzylamine in MeCN, followed by electron storage quantification upon K‐PHI(e^−^/H^+^) reaction with methyl viologen dichloride [13]. Varying light intensity (entries 1–3) revealed that an optical power of 100 mW cm^−^^2^ is sufficient for efficient conversion of TEA. At a lower intensity of 45 mW cm^−^^2^, the availability of photons is the factor limiting the yield of Et_2_NH (entry 2), while at a higher intensity of 360 mW cm^−^^2^, the reaction mixture is saturated with photons as the yield of Et_2_NH is the same as at 100 mW cm^−2^ (entry 3 vs. entry 1). Comparing the photocharging capabilities with PHIs, *N*_e_:*N*_hept_ values for melon‐type g‐CN (specific surface area 17 m^2^ g^−1^) and mesoporous graphitic carbon nitride (mpg‐CN, specific surface area 124 m^2^ g^−1^) are approximately 1:11 and 1:7, which we calculated from Et_2_NH yield of 9% and 14%, respectively (entry 4). Based on existing literature, K‐PHI and poly(triazine imide) (PTI) were synthesized [35, 37, 48]. Their performance in the triethylamine dealkylation reaction confirmed that changes in the cation can affect the overall electron storage capacity to some extent (entry 5), while the control group using PTI fully demonstrated the importance of PHI in the triethylamine dealkylation reaction (entry 6, see Figure S14 and Supplementary Discussion 6 for characterization of these materials and more details). Among multiple factors influencing the performance of materials in the dealkylation of TEA, these results underscore the critical role of the microporous 2D structure of PHIs, which outperforms the polymeric and mesoporous analogs lacking microporosity. Control experiments confirmed that both light irradiation and carbon nitride, the acceptor of hydrogen atoms, are crucial for the reaction (entries 7 and 8).

**TABLE 1:** Selected conditions of photochemical triethylamine dealkylation.

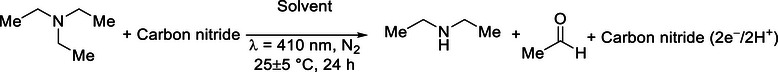				
Entry	TEA (mmol)	Carbon nitride	Yield (%)^a^	*N*_e_:*N*_hept_ ^b^
1	0.05	Na‐PHI	24	1:4.03
		H‐PHI	27	1:4.0
2^c^	0.05	Na‐PHI	10	1:9.62
		H‐PHI	9	1:12.1
3^d^	0.05	Na‐PHI	27	1:3.58
		H‐PHI	30	1:3.59
4	0.05	g‐CN	9	1:11.1
		mpg‐CN	14	1:7.09
5	0.05	K‐PHI	23	1:4.12
6	0.05	PTI^e^	12	‒^f^
7^g^	0.05	None	0	N/A
8^h^	0.05 0.05	Na‐PHI	0	N/A
		H‐PHI	0	N/A
9	0.05	H‐PHI 320 mg	99	1:16.7
10	0.5	H‐PHI 320 mg	10	1:15.8

Standard reaction conditions: triethylamine (TEA, specific amount), carbon nitride (20 mg), MeCN (2 mL), blue light (410 nm, 100 mW cm^−2^), reaction time (24 h), N_2_. N/A—data is not available.

^a^ Yields were determined from ^1^H NMR spectra using 1,3,5‐trimethoxybenzene as an internal standard.

^b^ *N*_e_:*N*_hept_ values were calculated according to the equation: $N_{e}:\;N_{\mathrm{hept}}=\frac{2\times n_{Et_{2}\mathrm{NH}}\times M_{w}}{k_{c}\times 10^{6}}$ Where $n_{Et_{2}\mathrm{NH}}$ *—*amount of diethylamine formed in the reaction, mmol. *k_c_*—number of heptazine units in the unit cell. *M*_w_ *—*molar mass of the unit cell, g mol^−1^. See Supplementary Method 1.6 for more details.

^c^ Blue light (410 nm, 45 mW cm^−2^).

^d^ Blue light (410 nm, 360 mW cm^−2^).

^e^ Reaction condition: triethylamine (0.05 mmol), MeCN (2 mL), blue light (365 nm, 100 mW cm^−2^), reaction time (24 h), N_2._

^f^ Given that the structure of PTI differs from the PHI structure, *N*_e_:*N*_hept_ parameter was not calculated.

^g^ Reaction condition: triethylamine (0.05 mmol), MeCN (2 mL), blue light (410 nm, 100 mW cm^−2^), reaction time (24 h), N_2_.

^h^ Reaction condition: triethylamine (0.05 mmol), carbon nitride (20 mg), MeCN (2 mL), reaction time (24 h), N_2_.

**FIGURE 3:**
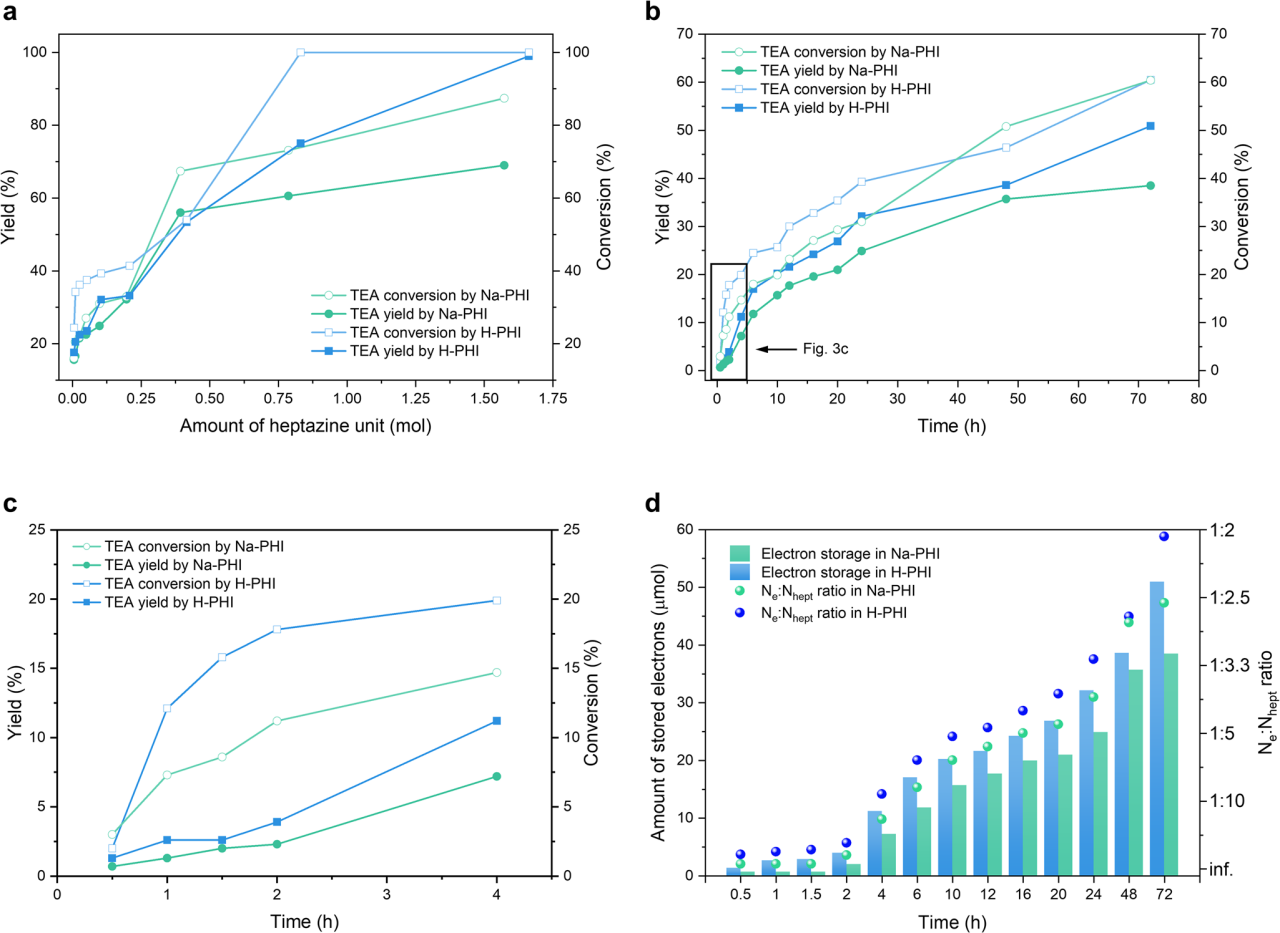
TEA dealkylation study. (a) Dependence of TEA conversion and Et_2_NH yield on the number of heptazine units in the given mass of PHI at the beginning of the photochemical reaction. Experimental conditions: TEA (0.05 mmol), carbon nitride (specific amount), MeCN (2 mL), blue light (410 nm, 100 mW cm^−2^), reaction time (24 h). (b) Dependence of TEA conversion and Et_2_NH yield on the duration of the photochemical reaction. Experimental conditions: TEA (0.05 mmol), carbon nitride (20 mg), MeCN (2 mL), blue light (410 nm, 100 mW cm^−2^). Each point corresponds to a separate experiment. (c) TEA dealkylation performed for 0.5–4 h. See (b) for the full dataset. Each point corresponds to a separate experiment. (d) Correlation between the number of stored electrons, electron density in photocharged PHI, and reaction time. Experimental conditions: TEA (0.05 mmol), carbon nitride (20 mg), MeCN (2 mL), blue light (410 nm, 100 mW cm^−2^).

Considering the chemical and crystal structure of PHIs [9, 49], there are 4.9 and 5.2 mmol of heptazine units in 1 g of Na‐PHI and H‐PHI, respectively (Table S5). We conducted a series of dealkylation experiments by varying the mass of H‐PHI and Na‐PHI while keeping the TEA amount constant at 0.05 mmol. Figure 3a shows TEA conversion and Et_2_NH yield depending on the mass of H‐PHI and Na‐PHI, and, therefore, the number of heptazine units (mol) employed in the reaction. The dependencies are nonlinear. These findings agree with the previous observations that conversion of benzylamine and the yield of N‐benzylidenebenzylamine, and N,N‐dibenzyl‐1,2‐diphenylethanediamine scale nonlinearly with the mass of carbon nitrides, that is, the number of heptazine units in the sample that are available as acceptors of electron‐proton pairs [22]. Notably, H‐PHI exhibits higher TEA conversion and Et_2_NH yield than Na‐PHI. According to the reaction stoichiometry, conversion of one TEA equivalent requires at least two equivalents of heptazine units. Therefore, theoretically, in the photochemical experiment with 0.05 mmol TEA, at least 0.1 mmol of heptazine units or 21 and 19 mg of Na‐PHI and H‐PHI are required to achieve complete conversion of TEA and 100% yield of Et_2_NH. However, our experiment achieved full conversion of TEA and 99% yield of Et_2_NH using 320 mg of H‐PHI (Table 1, entry 9). Such a significantly higher excess of heptazine units than required by the reaction stoichiometry might be due to kinetic or thermodynamic factors, or both. Using 0.5 mmol of TEA and 320 mg of H‐PHI (Table 1, entry 10) while maintaining other conditions the same, we obtained Et_2_NH with proportionally lower yield compared to the 0.05 mmol scale. These results suggest that the *N*_e_:*N*_hept_ ratio, approximately 1:16 (entries 9 and 10), achieved in these experiments is the same regardless of the amount of TEA.

The study of TEA conversion and Et_2_NH yield on the duration of the photochemical experiment (Figure 3b) revealed an initial induction period (Figure 3c). The concentration‐time profile for Et_2_NH follows apparent second‐order kinetics. The average photocharging rate was calculated based on the number of e^−^/H^+^ pairs stored in PHI over successive 4‐h intervals (Table S6). The highest rate for H‐PHI was 0.0465 µmol min^−^^1^ in the first 4 h, compared to 0.0301 µmol min^−^^1^ for Na‐PHI. These results, in conjunction with earlier analyses, suggest that the lower *E*_b_ value in H‐PHI is a key factor driving its more efficient photocharging. The reduced *E*_b_ facilitates greater surface potential change upon photoexcitation, thereby enhancing both the rate of TEA conversion and electron storage.

We extended the study to a broader scope of tertiary aliphatic amines (Table 2). Using H‐PHI as a heterogeneous hydrogen atom acceptor, (*n*Bu)_2_NH was obtained from (*n*Bu)_3_N in 94% yield (entry 2). Increasing the loading of (*n*Bu)_3_N 10 times to 0.5 mmol while maintaining the mass of H‐PHI the same (320 mg) led to the proportional decrease of (*n*Bu)_2_NH yield (entry 3), which is consistent with the similar experiment conducted using TEA (Table 1, entry 10). This observation confirmed that the number of e^−^/H^+^ pairs stored in H‐PHI remained comparable to those observed under the standard conditions (Table 1, entry 10 and Table 2, entries 1, 2), suggesting consistent photocharging behavior across different reaction scales. When using *N*,*N*‐diisopropylethylamine (DIPEA) as the substrate, H‐PHI selectively removes the ethyl group, leading to the products di*iso*propylamine and *iso*propylethylamine in a 2.9:1 ratio (entry 4). This selectivity can be rationalized by steric hindrance and a preferable formation of an intermediate of DIPEA one‐electron oxidation, followed by H‐atom abstraction from the ethyl's CH_2_‐group (Supplementary Discussion 3). The apparent quantum yields (AQY) for the dealkylation of TEA, Bu_3_N, and DIPEA by H‐PHI at 410 nm are 0.101%, 0.098%, and 0.095%, respectively (see Supplementary Method 1.7 for the calculation formula). In contrast, only modest yields were obtained in the dealkylation of *N*,*N*‐diethylbenzylamine and *N*,*N*‐dibutylbenzylamine, possibly due to reduced substrate reactivity or steric hindrance (entries 5 and 6).

**TABLE 2:** Comparison of H‐PHI with molecular sensitizers in the dealkylation of tertiary aliphatic amines.

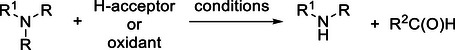				
Entry	H‐acceptor or oxidant	Tertiary amine	Major product yield (%)^a^	Minor product yield (%)^a^
1	H‐PHI	Et_3_N	Et_2_NH 99%	N.D.
2	H‐PHI	(*n*Bu)_3_N	(*n*Bu)_2_NH 94%	N.D.
3^b^	H‐PHI	(*n*Bu)_3_N	(*n*Bu)_2_NH 10%	N.D.
4	H‐PHI	DIPEA	(*i*Pr)_2_NH 66%	*i*PrEtNH 23%
5	H‐PHI	BnEt_2_N	Et_2_NH 30%	BnEtNH 7%
6	H‐PHI	Bn(*n*Bu)_2_N	(*n*Bu)_2_NH 20%	Bn(*n*Bu)NH 3%
7^c^	MV^2+^ 2PF_6_^−^	Et_3_N	Et_2_NH 62%	N.D.
8^c^	Benzophenone	Et_3_N	Et_2_NH 33%	N.D.
9^c^	MV^2+^ 2PF_6_^−^	(*n*Bu)_3_N	(*n*Bu)_2_NH 16%	N.D.
10^c^	Benzophenone	(*n*Bu)_3_N	(*n*Bu)_2_NH 35%	N.D.
11^c^	MV^2+^ 2PF_6_^−^	DIPEA	(*i*Pr)_2_NH 12%	*i*PrEtNH 2%
12^c^	Benzophenone	DIPEA	N.D.	N.D.

Reaction conditions: tertiary aliphatic amine (R_3_N, 0.05 mmol), carbon nitride (320 mg), MeCN (2 mL), blue light (410 nm, 100 mW cm^−2^), reaction time (24 h).

^a^ Yields were determined from ^1^H NMR spectra using 1,3,5‐trimethoxybenzene as an internal standard.

^b^ (*n*Bu)_3_N (0.5 mmol), carbon nitride (320 mg), MeCN (2 mL), blue light (410 nm, 2×100 mW cm^−2^), reaction time (24 h).

^c^ Tertiary aliphatic amine (R_3_N, 0.05 mmol), hydrogen atom acceptor or oxidant (2 equiv.), H_2_O (2 equiv.), MeCN (2 mL), blue light (410 nm, 100 mW c^−2^), reaction time (24 h).

Electronically excited state of methylviologen bis(hexafluorophosphate) (MV^2+^2PF_6_^−^) [50] and benzophenone [51] can act as one‐electron oxidant and/or hydrogen atom acceptor. Their one‐electron reduction products are methylviologen radical and ketyl radical, respectively. We benchmarked the performance of H‐PHI versus these molecules. Two equivalents of MV^2+^2PF_6_^−^ versus tertiary amine, gave Et_2_NH and (*n*Bu)_2_NH in 62 and 16% yield (entry 7,9), respectively, while in the case of benzophenone, we obtained Et_2_NH and (*n*Bu)_2_NH in 33 and 35% yields (entry 8,10), respectively. Furthermore, when using DIPEA, MV^2+^2PF_6_^−^ gave di*iso*propylamine and *iso*propylethyl amine in a 6:1 ratio (entry 11). Therefore, the excited state of MV^2+^2PF_6_^−^ is more selective toward removing the ethyl group compared to H‐PHI. However, the combined yield of di*iso*propylamine and *iso*propylethylamine was 14%. Benzophenone failed to react with DIPEA (entry 12).

Owing to the chemical stability of H‐PHI, we recovered this material by exposing photocharged H‐PHI(2e^−^/2H^+^) to air, followed by treatment with organic solvents, aqueous solution of hydrochloric acid, deionized water, and drying. After seven consecutive cycles of H‐PHI recovery and reuse in the photochemical reaction, the yield of (*n*Bu)_2_NH remained above 90% (Figure S15a). To assess the material's stability, H‐PHI samples after the third and fifth cycles were analyzed by PXRD, UV–vis, and FTIR spectroscopy. According to PXRD analysis, the material's crystal structure remains apparently intact (Figure S15b), while FT‐IR spectra revealed no alteration of the chemical structure (Figure S15c), indicating the high stability of H‐PHI against photocorrosion [52]. UV–vis spectra of H‐PHI excluded photobleaching—the position of the absorption edge, at ∼450 nm, remains unchanged, while no additional spectral features evolved in the visible range (Figure 15d). Collectively, these data demonstrate that H‐PHI retains its structural and chemical integrity throughout the reaction cycles, supporting its role as a recyclable hydrogen acceptor in photochemical dealkylation of tertiary amines.

## 3 Investigation of the Mechanism of Triethylamine Dealkylation by Theoretical Modelling

**FIGURE 4:**
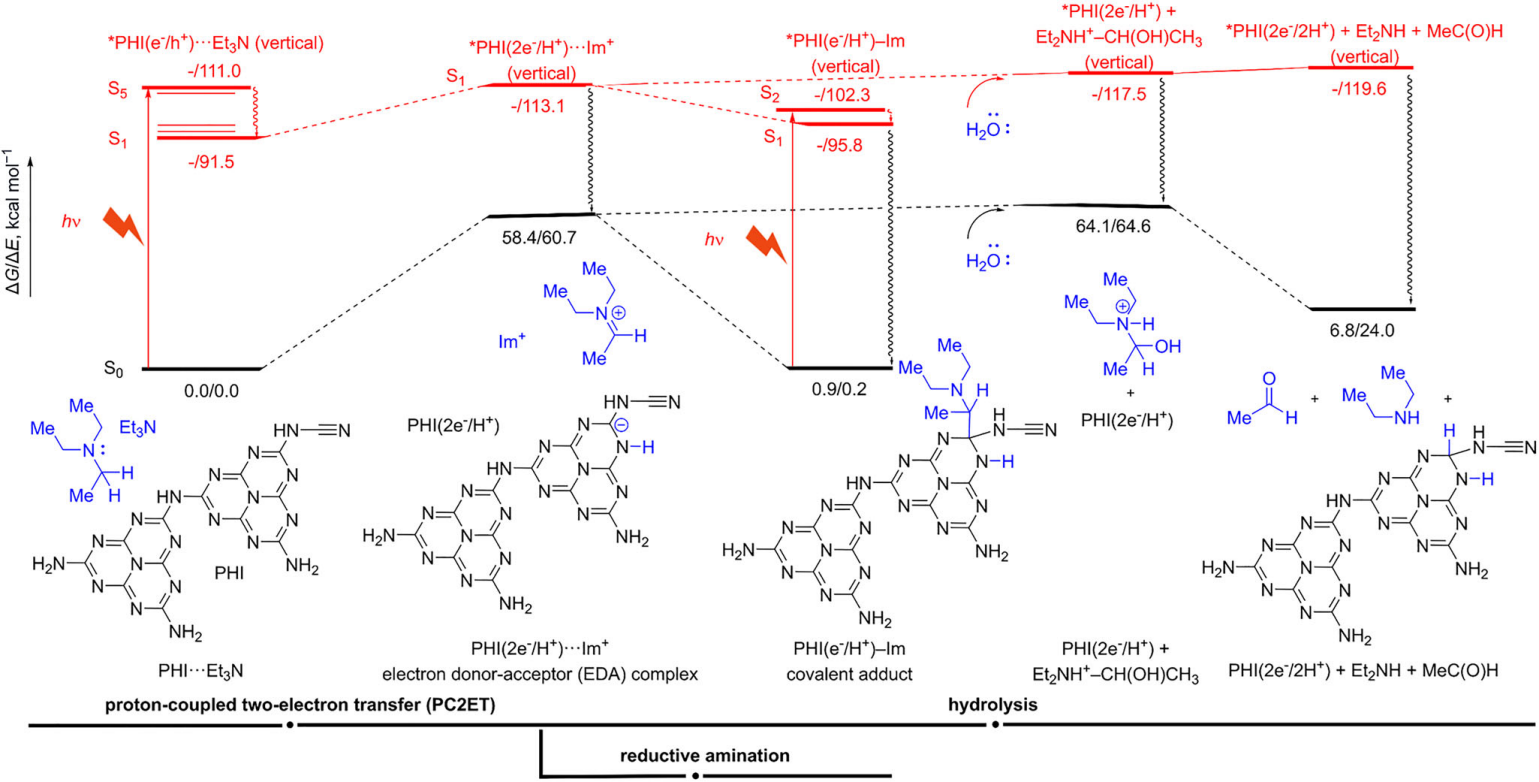
Results of modeling of photochemical TEA dealkylation reaction with H‐PHI. Calculations were performed with ωB97X‐D/def2‐SVP in implicit, acetonitrile solvent, with initial explorations performed with AIQM1 and AIQM2 methods. Excited‐state calculations were performed with TD‐ωB97X‐D/def2‐SVP in implicit solvent (acetonitrile) on the geometries optimized in the ground state.

We invoked theoretical investigations to shed light on the underlying processes and explain a nonlinear dependence of TEA conversion and Et_2_NH yield on the mass of PHIs (or the number of heptazine units employed in the photochemical experiments, Figure 3a). Due to the high complexity of the system, we adopted several simplifications in our modeling. In the first set of computational experiments, PHIs were modeled with two heptazine units (Figure 4). Due to the strong electron‐withdrawing nature of the CN groups present in PHI, they might play an important role in the oxidation of TEA; hence, we also included one of the cyanamide groups in one of the heptazine units. For simulations, we applied a combination of the methods, including the artificial intelligence‐enhanced quantum mechanical (AIQM) [53, 54], density functional theory (DFT), and semi‐empirical methods, as well as employed the Aitomia assistant [55] with AI agents for setting up some of the calculations (see Supplementary Methods 1.16). The results reported below use the DFT functional ωB97X‐D and include solvent effects, because acetonitrile is a polar solvent that can strongly influence electron transfer (ET) processes. Note that the range‐separated functional ωB97X‐D is known to often overestimate the excitation energies; however, it is more suitable for simulating electron transfer than non‐range‐separated functionals such as B3LYP. Hence, we use ωB97X‐D for qualitative investigation of the photochemical processes rather than targeting the best agreement with the experimental excitation energies. Due to the complexity of the systems, for modeling excited states, we have adopted a further approximation by performing time‐dependent (TD) DFT calculations on the ground‐state minima.

The modeling reveals a complex picture featuring several interesting phenomena (Figure 4). The photoreactions are driven by photoexcitation of the initial complex PHI···TEA to the first bright S_5_ state, which is expected to quickly nonradiatively relax to the S_1_ state, where the further photoprocesses proceed. The initial complex undergoes the proton‐coupled two‐electron transfer (PC2ET) [56] leading to the formation of the intermediate, electron donor‐acceptor (EDA) complex of PHI(2e^−^/H^+^)···Im^+^, where Im^+^ denotes iminium cation. In this EDA complex, the second electron is almost fully transferred to PHI as follows from the charge analysis: the charge on the Im moiety is +0.9 e, both in the ground and excited states (S_0_ and S_1_), showing that two electrons were transferred rather than one. We note that the alternative pathway via intersystem crossing would lead to the hydrogen‐atom transfer instead, because the analogous intermediate PHI(e^−^/H^+^)···Im^·^ in the triplet T_1_ state contains heptazinyl PHI(e^−^/H^+^) and α‐aminoalkyl radicals without any electron transfer (the calculations show that the Im moiety is neutral in the triplet intermediate). Hence, we ruled out the triplet pathway as the iminium radical would likely lead to different products.

On the singlet surfaces, after the formation of the EDA complex, we observe two competing photochemical reactions: one leading to the expected dealkylation product Et_2_NH and another to the potential by‐product, the covalent adduct of iminium cation with PHI. In the presence of water, the EDA complex can undergo hydrolysis to Et_2_NH via a cationic intermediate. Anionic PHI(2e^−^/H^+^) abstracts a proton from this intermediate, facilitating the hydrolysis and ultimately yielding Et_2_NH and neutral PHI(2e^−^/2H^+^) charged with two electrons and protons; one proton from Et_3_N attaches to nitrogen, while another from water attaches to carbon in PHI(2e^−^/2H^+^). Note that we could not find the PHI(2e^−^/2H^+^) with both hydrogens located on nitrogens that are lower in energy than PHI(2e^−^/2H^+^) with hydrogens attached to carbon and nitrogen. The final products are very high in energy in the excited state; hence, they might be formed after the system undergoes relaxation to the S_0_ surface at one of the steps after the EDA complex formation. Once in the ground state, the final products are endothermic as they are 6.8 kcal mol^−1^ above the reactants; the total electronic energy is 24.0 kcal mol^−1^ above the reactants. Hence, the product partially stores the photon energy.

The competing process to the dealkylation is the formation of the covalent adduct PHI(e^−^/H^+^)–Im from the EDA complex, that is, reductive amination of the PHI. In this process, the carbon in the iminium cation can readily recombine with the carbon in the anionic PHI(2e^−^/H^+^), forming a covalent bond and leading to the adduct. This adduct is endothermic by only 0.9 kcal mol^−1^ in the ground state. Hence, if the system undergoes relaxation to the ground state, it is very likely to get trapped in this covalent adduct. Indeed, this finding from the independent simulations was later confirmed by experimental observation of the mismatching mass balance (see Discussion below, and conversion and yield in Table S5). The covalent adduct, despite being more thermodynamically stable than the dealkylation product, is likely to undergo the reverse process of forming the EDA complex in the excited state, where it can be hydrolyzed into Et_2_NH. The product species Et_2_NH, acetaldehyde, and the photocharged sites in PHI(2e^−^/H^+^) (a heptazine unit bearing two electrons and one proton) can be spatially removed from each other, preventing them from reacting with each other, hence, shifting the equilibrium balance to the formation of the main product. Also, the electron/proton couples can migrate from the surface to the bulk of PHI(2e^−^/H^+^), further shifting the balance. When the covalent adduct ends up in the ground state, it can absorb another photon during the long photoirradiation, starting the cascade of the main reaction transformations via the EDA complex. This explains why the prolonged experiments lead to higher yields of the main product Et_2_NH without seeing an increase in the byproducts.

Overall, when dealkylation of TEA as such is concerned, this reaction is enabled photochemically employing PHI as an H_2_ acceptor. Strong signal in EPR spectra that earlier was assigned to heptazinyl radicals indicates that PHI does not store H_2_ molecules, but electron–proton couples, which may be released on demand in the form of H_2_ gas [10], or used to reduce O_2_ to H_2_O_2_ [57] or organic molecules in the dark [58].

**FIGURE 5:**
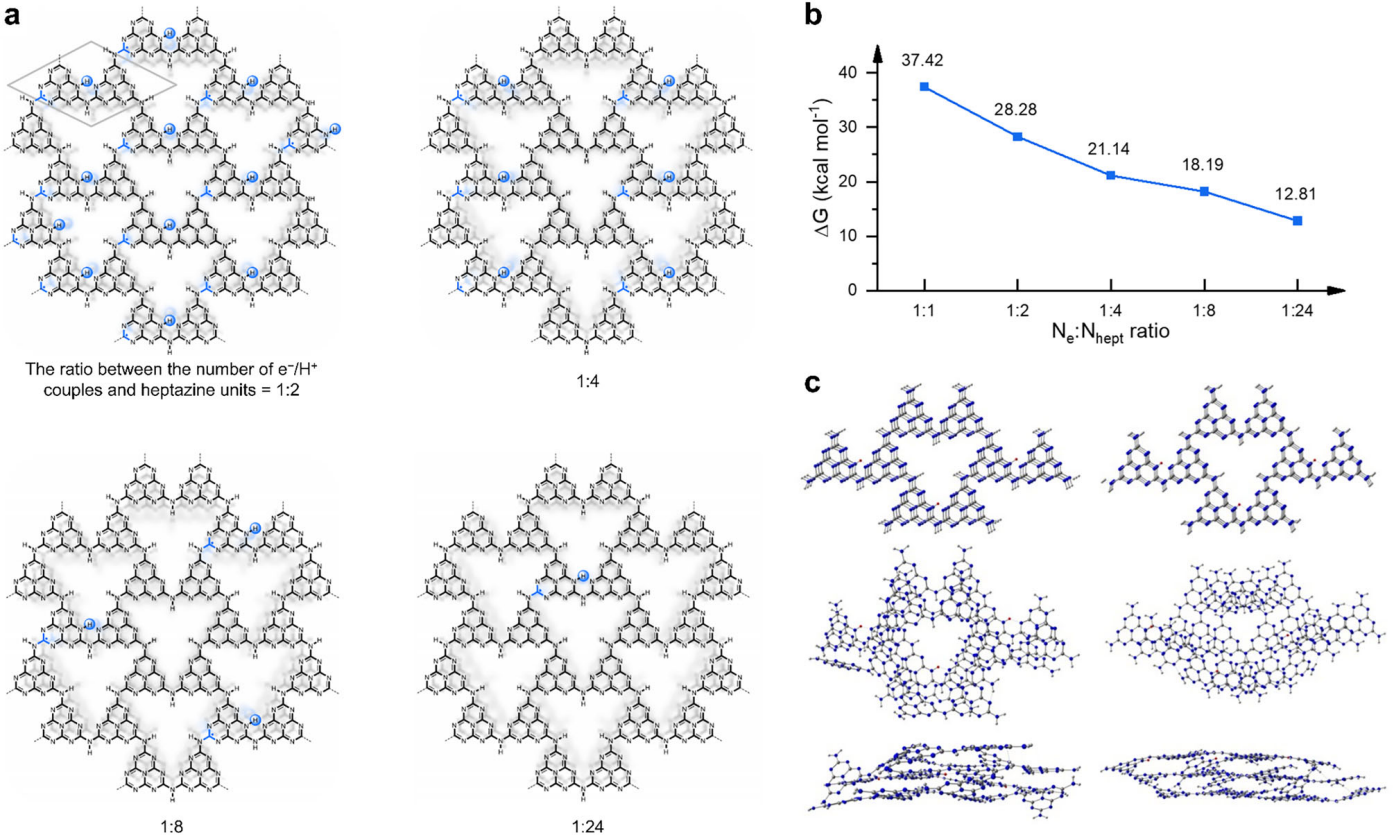
Results of H‐PHI(e^−^/H^+^) modelling featuring different *N*_e_:*N*_hept_ ratios. (a) Schematic illustration of the molar ratio between electron‐proton couples and heptazine units in H‐PHI(e^−^/H^+^). The molecular model of H‐PHI is shown in the gray line area. (b) Dependence of ΔG_0_ and ΔH_0_ of the photochemical reactions on the density of electron–proton couples in H‐PHI(e^–^/H^+^). (c) Structures of photocharged H‐PHI featuring *N*_e_:*N*_hept_ ratio 1:8 and e^–^/H^+^ couples stored on the surface and in the bulk.

The results of the reaction profile modeling assume a photochemical reaction between TEA and heptazine units of H‐PHI in a 1:2 molar ratio, with the *N*_e_:*N*_hept_ ratio of 1:1. Although in our modeling, two electrons ended up on the same heptazine unit, they are subsequently likely to migrate to different units. In a series of experiments, the *N*_e_:*N*_hept_ ratio is greater than 1:1 (Figure 3d), that is, statistically, an electron and a proton are stored over more than one heptazine unit (Figure 5a). There can be several factors affecting the ratio, which we elaborate on below.

One potential factor is that the proton‐coupled two‐electron transfer is facilitated by the presence of the electron‐withdrawing cyanamide groups. It might happen that the reaction proceeds fast until the heptazine units with cyanamide groups are depleted, that is, the *N*_e_:*N*_hept_ ratios might correlate with the fraction of heptazine units with cyanamide groups. The latter is, however, difficult to quantify experimentally.

Another factor is how the thermodynamics (Δ*H* and Δ*G*) of the dealkylation reaction are affected by the degree of PHI photocharging. To gain insights into this, we created a model of a single H‐PHI layer composed of 24 heptazine units. Due to the large size of the systems, we adopted the semi‐empirical GFN2‐xTB method. Given that the reaction between TEA and water gives Et_2_NH, acetaldehyde, and 2e^–^/2H^+^, we calculated Δ*H* and Δ*G* values of the reaction of this model with 1/2, 3/2, 3, 6, and 12 TEA molecules. Reaction of this number of TEA molecules with the H‐PHI model composed of 24 heptazine units yields an equal number of Et_2_NH and acetaldehyde molecules, and a photocharged H‐PHI(e^–^/H^+^) with the *N*_e_:*N*_hept_ ratios 1:24, 1:8, 1:4, 1:2, and 1:1, respectively. Δ*G* values of these dealkylation reactions scale reversibly with the extent to which H‐PHI is photocharged (Figure 5b). Thus, the reaction, which leads to the formation of a photocharged H‐PHI bearing one e^–^/H^+^ couple per heptazine unit, is more endothermic and endergonic compared to the reaction in which photocharged H‐PHI bears one e^–^/H^+^ couple per 24 heptazine units.

We assume that the initial photoredox process between a molecule of tertiary amine and a heptazine unit occurs on the surface of PHI. Therefore, 2 e^–^/H^+^ couples, which are released upon oxidation of one molecule of tertiary amine, are initially stored in heptazine units closer to the PHI particle surface. Given that PHIs are electron and ion conductors [32], and we estimated *N*_e_:*N*_hept_ ratios to be in the range 1:16–1:4 from the experimental data (Table 1), we assume migration of the 2 e^–^/H^+^ couples into the bulk of the PHI particle. To evaluate the driving force of this process, we built another structure that is composed of three H‐PHI layers. Each layer is composed of 8 heptazine units; that is, there are 24 heptazine units in the structure in total. Furthermore, we created two structures of photocharged H‐PHI, in which 3 e^–^/H^+^ couples were added (Figure 5c). These structures correspond to *N*_e_:*N*_hept_ ratio of 1:8. In the first instance of such a photocharged H‐PHI, the 3 e^–^/H^+^ couples were added to the top layer, that is, three heptazinyl radicals are located within the same top layer. In the second instance of the photocharged H‐PHI, 3 e^–^/H^+^ couples were added to the middle layer. Comparison of ΔG^0^ values of the dealkylation reaction that produces such photocharged H‐PHI structures indicates that migration of e^–^/H^+^ couples from the surface into the bulk is by 4.6 kcal mol^−1^ exergonic and, as such, occurs spontaneously. This driving force rationalizes the migration of e^–^/H^+^ couples and explains the ion conductivity of PHIs. Similar calculations but performed for a photocharged H‐PHI featuring *N*_e_:*N*_hept_ ratio 1:4, indicate that migration of e^–^/H^+^ couples from the surface into the bulk is by 15.9 kcal mol^−1^ endergonic (non‐spontaneous). In other words, e^–^/H^+^ couples tend to accumulate on the surface of the H‐PHI photocharged to a greater extent, if there are still enough surface heptazine units available for e^–^/H^+^ storage.

## 4 Conclusion

This work elucidates the electron storage capacity and active sites in poly(heptazine imide) (PHI) through photochemical dealkylation of tertiary amines. By introducing a quantitative descriptor, *N*_e_:*N*_hept_, and merging experimental and theoretical results, we reveal the structural and energetic parameters governing the photocharging process. Thus, we found that:

1. H‐PHI, characterized by a lower exciton binding energy, exhibits faster charge accumulation and greater recyclability than the Na‐PHI analog. As an advanced electron storage platform, the H‐PHI retained structural stability over seven rounds, which supports its use in recyclable photoinduced redox reactions.
2. Heptazine units in PHI do not function as independent photoredox sites, but photocharging of one heptazine affects the adjacent one, and makes its excited state, presumably, less oxidative. This explains the nonlinear dependence of tertiary amine conversion and secondary amine yield on the number of heptazine units available for the reaction.
3. When PHI is used as the only acceptor and storage medium of electron–proton couples, to achieve a complete conversion of the hydrogen atom donor, the molar ratio of heptazine units to the reactant should be >4:1, for practical purposes. Naturally, this ratio can be <1:1 if a sacrificial oxidant is employed, which allows for recovery of PHI(e^−^/H^+^) in the photocatalytic cycle.

These findings advance our understanding of charge storage in carbon nitrides and offer a general approach for developing metal‐free, photoactive semiconductors with tailored energy storage capabilities and photochemical transformations of organic molecules.

## Conflicts of Interest

The authors declare no conflicts of interest.

## Supporting information




**Supporting File 1**: The authors have cited additional 45 references within the Supporting Information.

## Data Availability

The data that support the findings of this study are available from the corresponding author upon reasonable request.
