# Supplementary material for: Photochemical N‐dealkylation of Tertiary Amines Coupled With Photocharging of Poly(Heptazine Imides)
Source: Angew Chem Int Ed Engl. 2026 Feb 4;65(11):e22677. doi: 10.1002/anie.202522677 (PMC12970505; doi:10.1002/anie.202522677)
Supplement: Supplementary file 1 — Supporting File 1: The authors have cited additional 45 references within the Supporting Information. [file ANIE-65-e22677-s001.pdf]

Supporting information

Photochemical N-dealkylation of Tertiary Amines Coupled with Photocharging of Poly(Heptazine Imides)

Jingru Zhuang[a], Quanhao Zhang[b], Chong Wang[a], Tao Yao[a], Cheuk Lok Cheng[a], Pavlo O. Dral*[b][c][d], Oleksandr Savateev*[a]

[a] Department of Chemistry, The Chinese University of Hong Kong, Shatin, New Territories, Hong Kong, China.

[b] State Key Laboratory of Physical Chemistry of Solid Surfaces, Department of Chemistry and Chemical Engineering, College of Chemistry and Chemical Engineering, and Fujian Provincial Key Laboratory of Theoretical and Computational Chemistry, Xiamen University, Xiamen 361005, Fujian, China.

[c] Institute of Physics, Faculty of Physics, Astronomy, and Informatics, Nicolaus Copernicus University in Toruń, ul. Grudziądzka 5, 87-100 Toruń, Poland.

[d] Aitomistic, Shenzhen 518000, China.

Corresponding Authors

*Email: dral@xmu.edu.cn (P. O. Dral)

*Email: oleksandrsavatieiev@cuhk.edu.hk (O. Savateev)

Supplementary Methods

1.1. Preparation of reagents and materials

Melamine (98%) was purchased from BDH; tributylamine (99%) was purchased from Sigma-Aldrich; triethylamine (>99%) was purchased from International Laboratory; N,N-diethylbenzylamine (97%) was purchased from Leyan; diisopropylethylamine (99.5%) was purchased from J&K; sodium chloride (>99%) was purchased from Dieckmann; 1,3,5,-trimethoxybenezene (98%) was purchased from Macklin; acetonitrile, tetrahydrofuran, and methanol was purchased from RCI Labscan in gradient HPLC grade.

These chemicals have been used as received without purification.

Na-PHI (S_{SA} = 24.3 m^2 g^{-1}) has been prepared by modifying the reported procedure.[1] Melamine (1 g) was thoroughly grinded with NaCl (10 g) in a mortar. The resultant mixture was transferred into a porcelain crucible which was covered with a lid. The crucible was placed in a muffle furnace and heated to 873 K with a heating rate of 2.3 K min^{-1}, held at 873 K for 4 hours. After cooling to room temperature spontaneously, the crude product was removed from the crucible, washed with deionized water (1 L), isolated by filtration, then thoroughly washed with deionized water on the filter (1 L) and dried in an oven for 16 hours.

H-PHI (S_{SA} = 30.8 m^2 g^{-1}) has been prepared by modifying the reported procedure.[2] To a solution of HCl (50 mL, 0.1 mol L^{-1}) was added Na-PHI (1 g). The mixture was maintained in a sonication bath for 2 hours. After stirring at room temperature for 48 hours, the resulting suspension was filtered, filtrate was washed with deionized water (~500 mL) until pH = 7 and dried in an oven for 16 hours.

K-PHI has been prepared by modifying the reported procedure.[3] g-CN (1 g) was thoroughly ground with KCl (5.5 g) and LiCl (4.5 g) in a mortar. The resultant mixture was transferred into a porcelain crucible and covered with a lid. The crucible was placed in a tube furnace, heated to 873 K with a heating rate of 2.3 K min^{-1}, held at 873 K for 4 hours, under N_2 atmosphere (200 mL min^{-1}). After cooling to room temperature spontaneously, the crude product was removed from the crucible, washed with deionized water (1 L), isolated by filtration, then thoroughly washed with deionized water on the filter (1 L) and dried in an oven for 16 hours.

PTI has been prepared by modifying the reported procedure.[4] g-CN (1 g) was thoroughly ground with KCl (5.5 g) and LiCl (4.5 g) in a mortar. The resultant mixture was transferred into a porcelain crucible and covered with a lid. The crucible was placed in a tube furnace, heated to 873 K with a heating rate of 2.3 K min^{-1}, held at 873 K for 8 hours, under N_2 atmosphere (200 mL min^{-1}). After cooling to room temperature spontaneously, the crude product was removed from the crucible, washed with deionized water (1 L), isolated by filtration, then thoroughly washed with deionized water on the filter (1 L) and dried in an oven for 16 hours.

1.2. N_2 physisorption

Measurements have been conducted on Anton Paar Autosorb 6100 physisorption station and

the data have been processed in Anton Paar kaomi software. Semiconductors have been degassed in vacuum at 353 K for 1 h and 423 K for further 14 h prior the measurements. N_2 sorption isotherms have been acquired at 77 K. Surface area has been determined from the absorption branch of N_2 Sorption isotherms in the range of P/P_0 values $0.025<P/P_0<0.99$.

1.3. NMR spectroscopy

^{1}H NMR and ^{13}C NMR spectra were recorded on Bruker AVANCE III HD 500 MHz (at 500 MHz for Protons, 125 MHz for Carbon-13). Chemical shifts are reported in ppm versus solvent residual peak: $CDCl_3$ 7.26 ppm (^{1}H NMR), 77.16 ppm (^{13}C NMR).

1.4. Irradiance measurements and light source

Irradiance of the LED modules was measured using Thor labs PM400 Optical Power and Energy Meter equipped with the integrating sphere S142C. Unless specified, irradiance of LED modules was measured at 4 cm distance: 410 nm, 100±0.5 mW cm^{-2}.

1.5. Measurement of exciton binding energy (E_b) of the synthesized samples

The E_b values were obtained from the following equation upon fitting the experimental data:[5]

$$I(T) = \frac{I_0}{1+A\,exp^{(\frac{-E_b}{k_B T})}} \quad (1)$$

where I_0 – PL intensity extrapolated to 0 K; T – temperature, K; k_B – Boltzmann constant, 1.381×10^{-23} J K^{-1}.

1.6. Measurement of the number of electrons stored in semiconductors

The method was adapted from the literature.[6] The total specific number of heptazine units in carbon nitride material (in mmol g^{-1}) was calculated according to the equation:

$$N_{total} = \frac{k_c \times 10^6}{M_w} \quad (2)$$

where k_c – number of heptazine units in the unit cell. For Na-PHI and H-PHI, k_c = 2. M_w – molar mass of the unit cell, g mol^{-1}. For Na-PHI, M_w = 407 g mol^{-1}; H-PHI, M_w = 385 g mol^{-1}; K-PHI, M_w = 423 g mol^{-1}.

According to the proposed mechanism, the formation of one mole of diethylamine would lead to two moles of electron/proton couples stored in the carbon nitride material. Total specific amounts of electron/proton couples stored in the carbon nitride material (in mmol) were calculated from the equation:

$$\delta = 2 \times n_{Et_2NH} \quad (3)$$

where n_{Et_2NH} – amount of diethylamine formed in the reaction, mmol.

1.7. Measurement of apparent quantum yield (AQY) for triethylamine dealkylation

AQY was calculated using the following equation:[7]

$$AQY\ (\%) = \frac{N_e}{N_p} \times 100\% = \frac{\frac{2 \times M \times N_A}{S \times P \times t}}{h \times \frac{c}{\lambda}} \times 100\% = \frac{2 \times M \times N_A \times h \times c}{S \times P \times t \times \lambda} \times 100\% \quad (4)$$

where N_e – total number of transferred electrons, N_p – number of incident photons; M – amount of product formed in the specific dealkylation, mol; N_A – Avogadro constant, 6.022×10^{23} mol^{-1}; h – Planck constant, 6.626×10^{-34} J s; c – speed of light, 3×10^8 m s^{-1}; S – the irradiation area, 3.3 cm^2 (see Supplementary Fig. 13); P - intensity of irradiation light, W cm^2; t – reaction time, s; λ – wavelength of the incident light, m.

1.8. X-ray diffraction

Powder X-ray diffraction (PXRD) was acquired using a Rigaku SmartLab with Cu-Kα (1.54 Å) emission and a scanning range between 5° and 80° (step of 0.01°)

1.9. Fourier-transform infrared

Fourier-transform infrared (FT-IR) spectra was recorded on Thermo Nicolet iS10 mid-FTIR spectrometer through KBr tableting method.

1.10. UV-vis diffuse reflectance spectra

UV-vis diffuse reflectance spectra (DRS) were conducted on Agilent Cary 100 spectrometer.

1.11. X-Ray photoelectron spectroscopy

X-Ray photoelectron spectroscopy was conducted on Kratos AXIS Supra+.

1.12. Electron paramagnetic resonance

Electron paramagnetic resonance (EPR) data was recorded on CIQTEK EPR200-Plus spectrometer using a high-sensitivity resonator ER4119HS (with a grid on the front side). The powder measurements were made in air atmosphere. Unless otherwise stated, the *in-situ* irradiation was performed using Xe lamp (300 W with a 410 nm band pass filter).

1.13. Temperature-dependent photoluminescence

Temperature-dependent photoluminescence was conducted on Edinburgh FLS980. Temperature ranging from 90 K to 300 K.

1.14. Semiconductor recycling and stability study

A 4-mL screw-caped glass vial was charged with a semiconductor (320 mg), tributylamine (0.05 mmol), MeCN (2 mL) and magnetic stir bar. The mixture was degassed three times *via* freeze-pump-thaw procedure and refilled with N_2. The mixture was stirred at room temperature

under irradiation with blue LED (410 nm, 100±0.5 mW cm^{-2}). After 24 h of irradiation, the reaction mixture was transferred to a centrifuge tube and centrifuged (13 300 rpm, 5 min). The supernatant layer was removed, the precipitate was washed by ethyl acetate, isopropyl alcohol. The precipitate was then dissolved in HCl (18 mL, 0.1 M) and maintained in a sonication bath for 1 h. Suspension was stirred at room temperature for 16 h and filtered. The resulting precipitate was washed with deionized water until pH = 7 and dried in oven at 333 K for 16 h. The dried semiconductor has been used for the next run without any additional purification.

1.15. A general procedure of dealkylation

For non-volatile reagents and products:

A 4-mL screw-caped glass vial was charged with photocatalyst, R_3N (0.05 mmol), MeCN (2 mL) and magnetic stir bar. The mixture was degassed three times *via* freeze-pump-thaw procedure and refilled with N_2. The mixture was stirred at room temperature under irradiation with blue LED (410 nm, 100±0.5 mW cm^{-2}) for 24 h. After irradiation was stopped, the reaction mixture was transferred into a 2 mL Eppendorf tube and centrifuged (13 300 rpm, 5 min). The supernatant layer was separated, and the precipitate semiconductor was washed with CH_2Cl_2 (3 x 2 mL) followed by centrifugation. A solution of 1,3,5-trimethoxybenzene in $CDCl_3$ (0.5 mL, 0.1 M) was then added to the combined organic phases. The resultant solution was concentrated *in vacuo*. The residue was dissolved in $CDCl_3$ (0.9 mL) and analyzed by ^1H NMR.

For volatile reagents and products:

A 4-mL screw-caped glass vial was charged with photocatalyst, R_3N (0.05 mmol), MeCN (2 mL) and a magnetic stir bar. The mixture was degassed three times *via* freeze-pump-thaw procedure and refilled with N_2. The mixture was stirred at room temperature under irradiation with blue LED (410 nm, 100±0.5 mW cm^{-2}) for 24 h. After irradiation was stopped, the reaction mixture was transferred into a 2 mL Eppendorf tube and centrifuged (13 300 rpm, 5 min). The supernatant layer (0.5 mL) was transferred into an NMR tube. A solution of 1,3,5-trimethoxybenzene in $CDCl_3$ (0.5 mL, 0.1 M) was added to the reaction mixture. The composition of the mixture was analyzed by ^1H NMR.

1.16. Computational details

All the calculation results with the XYZ coordinates, energies, and Jupyter notebooks with the analysis are openly available at https://github.com/dralgroup/phi.

We have performed extensive explorations with AIQM methods: AIQM1[8] and AIQM2[9]. While these methods provide excellent speed and accuracy for many types of simulations, particularly those in gas phase with neutral, closed-shell species in their ground state, these particular systems pose specific challenges such as: 1) many species are charged or radicals, 2) many processes are occurring on electronically excited surfaces, 3) solvent effects can be very important for charge separated species. AIQM1 has no good implementation of implicit solvent effects, while AIQM2 is inapplicable to excited-state simulations. Hence, for the discussion in the main text, after initial exploration with the AIQM methods, we have also employed DFT

methods.

Most of the calculations were performed with the MLatom program[10], while the final DFT calculations reported in the main text were performed with Gaussian 16[11]. The calculations were performed, and their analysis was assisted with Aitomistic Lab, providing a web interface for computational chemistry simulations on the group's high-performance computing cluster. Aitomistic Lab is equipped with Aitomia's AI agents assisting in performing simulations autonomously.[12]

The interface to the xtb program[13] was used for the GFN2-xTB* (GFN2-xTB without dispersion corrections)[14] method, which is also required by the AIQM2 approach. In the AIQM1[8] calculations, ODM2* contributions (ODM2[15] with removed dispersion interactions) were calculated with the MNDO program[16]. The D4 dispersion corrections[17] present in the AIQM1 and AIQM2 methods were calculated via the interface to the dftd4 program[18]. Neural network calculations in AIQM1 and AIQM2 were performed by generating the descriptors via the interface to the TorchANI package[19]. The interface to the Gaussian 16 program suite[11] was used as an engine for the optimizations of minima and frequency calculations. The Gaussian program was also used for the DFT calculations with the ωB97X-D[20]/def2-SVP[21-24] and B3LYP[25-26]/6-31G*[27-28] methods. In the calculations with ωB97X-D/def2-SVP, we used the polarizable conductor solvent model (C-PCM)[29-30] implicit solvent model. For excited state simulations, we used the time-dependent (TD)[31-38] DFT theory approach. The charge distributions were calculated using the Mulliken[39] atomic charges.

1.17. Photoelectrochemical measurement

Photoelectrochemical measurements were performed on a Chenhua CHI660E electrochemical workstation equipped with a standard three-electrode system. The Ag/AgCl and Pt sheet were used as the reference electrode and counter electrode, respectively. The working electrode was prepared on an ITO glass, which was thoroughly cleaned by sonication in ethanol for a duration of 0.5 h. A quantity of 5 mg of the samples was dispersed in 0.8 mL of acetonitrile, following which the mixture was sonicated for 0.5 h. Thereafter, 10 μL of the resulting slurry mixture was deposited onto the pre-cleaned ITO glass. The 0.2 M Na_2SO_4 solution was selected as the electrolyte without additive (pH = 7). Electrochemical impedance spectroscopy and Mott-Schottky measurement were conducted in the absence of irradiation. The transient photocurrent response experiment was measured by on/off irradiation cycles.

Supplementary Discussion 1

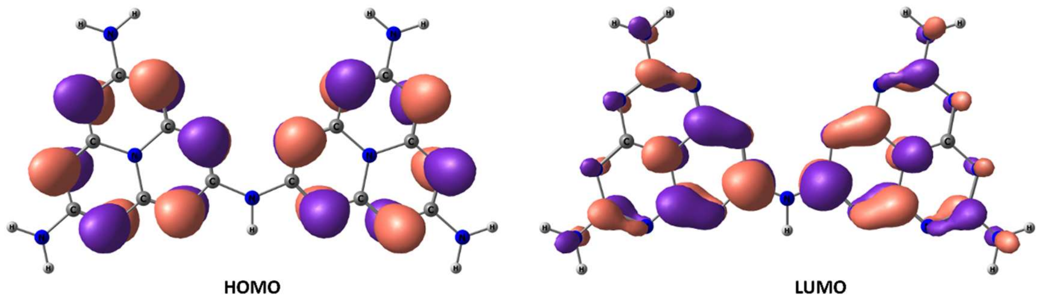

Poly(heptazine imide) (PHI) was modelled as a dimer in the singlet state to calculate its frontier orbitals. The result illustrates that HOMO, or the valence band in PHI, is localized on nitrogen atoms, and LUMO, the conduction band, is on carbon atoms. The results are obtained by using B3LYP as a DFT functional, 6-31G* as the basis set. Blue spheres represent nitrogen, gray ones carbon, and light gray ones hydrogen atoms. Results are consistent with the literature.[40-41]

Supplementary Discussion 2

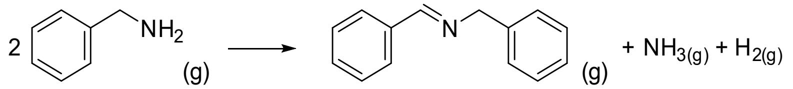

$\Delta H°$ = 10.2 kcal mol^{-1}
$\Delta G°$ = 2.7 kcal mol^{-1}

A reaction of condensation of two benzylamine molecules that gives *N*-benzylidenebenzylamine, NH_3, and H_2 in a gas phase is 11.0 kcal mol^{-1} endothermic at AIQM1, which is known[42-43] to provide accurate thermochemistry approaching chemical accuracy for gas-phase calculations of neutral, closed-shell organic molecules.

Supplementary Discussion 3

Regarding the selectivity of DIPEA dealkylation, we rationalize the results as follows. After proton-coupled two-electron transfer (PC2ET) takes place between the reactant and the PHI electronically-excited state, DIPEA is converted into iminium cation **1** or **2**, which is then hydrolyzed to produce acetone and isopropylethylamine **3** or diisopropylamine **4**, respectively:

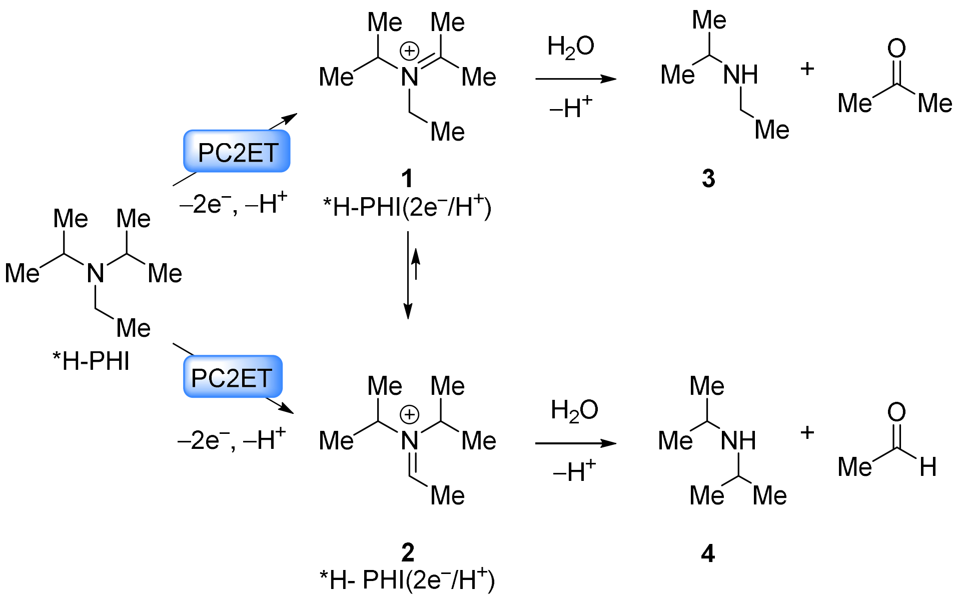

There are two aspects that could explain the higher selectivity of di-iso-propylamine formation from DIPEA:

1) Steric hindrance of the C–H bond next to the nitrogen atom. Ethyl's CH_2-group is more accessible for PC2ET by the electronically-excited H-PHI and MV^{2+} $2PF_6^-$ compared to that of the isopropyl CH-group. Therefore, intermediate **2** forms faster than intermediate **1**.

2) Equilibrium between intermediate **1** and **2** complexed with photoexcited *H-PHI(2e⁻/H⁺). If equilibrium is shifted towards the complex with intermediate **2**, hydrolysis of this species results in a higher yield of di-isopropylamine.

Considering that the selectivity of DIPEA dealkylation depends on the structure of sensitizer, faster PC2ET from a less sterically-hindered ethyl group to electronically-excited H-PHI and MV^{2+} $2PF_6^-$ is more plausible.

Supplementary Discussion 4

To further extend the application of photocharging, Na-PHI has been used to test the outdoor reaction of Bu_3N dealkylation due to its better absorption in the visible range. The "photochromic" phenomenon can be clearly seen after the reaction, indicating that electron-proton couples were stored in Na-PHI. After separating the reaction solution under ambient conditions, H_2O (2 mL) was added to the precipitate. After 30 min, the resultant mixture was separated, the supernatant layer was filtered, and analyzed by UV-vis; we found that 1 μmol H_2O_2 was formed (Supplementary Fig. 14).

Supplementary Discussion 5

In the EPR tests, the sample was added directly to the EPR tube, as detailed in Supplementary Method 1.12. The EPR tube was not degassed, and all reactions proceeded in an air atmosphere. In the initial reversible behavior test, we assumed that photoelectrons generated under light excitation would react with atmospheric oxygen to form superoxide anion radicals, which would initially suppress the signal. After equilibrium was reached, the signal from the photoelectrons increased over time.

Supplementary Discussion 6

To test the applicability of the structure-property relationships discussed in the main text to other materials, we synthesized and evaluated carbon nitride derivatives potassium poly(heptazine imide)(K-PHI) and the poly(triazine imide) (PTI) for structural comparison, using methods described in the literature, while the characterization results in (Supplementary Fig. 17) are consistent with the literature report.[3-4] These materials were tested under the same photocatalytic conditions in N-dealkylation of triethylamine as Na-PHI and H-PHI (Supplementary Table 8).

The photocharging and reaction trends of K-PHI were consistent with those PHI series. However, PTI showed a significant reduction in the production of Et_2NH. Given the unique nature of its structure, this work does not explore its charge storage density or the utilization of its structural units.

In summary, these observations support the exploration of the effect of cation alteration on photocharging efficiency in our work, and also demonstrate that PHI, with its microporous and ionic structure, plays a crucial role in stabilizing electron/proton (e^-/H^+) pairs.

Supplementary Figures

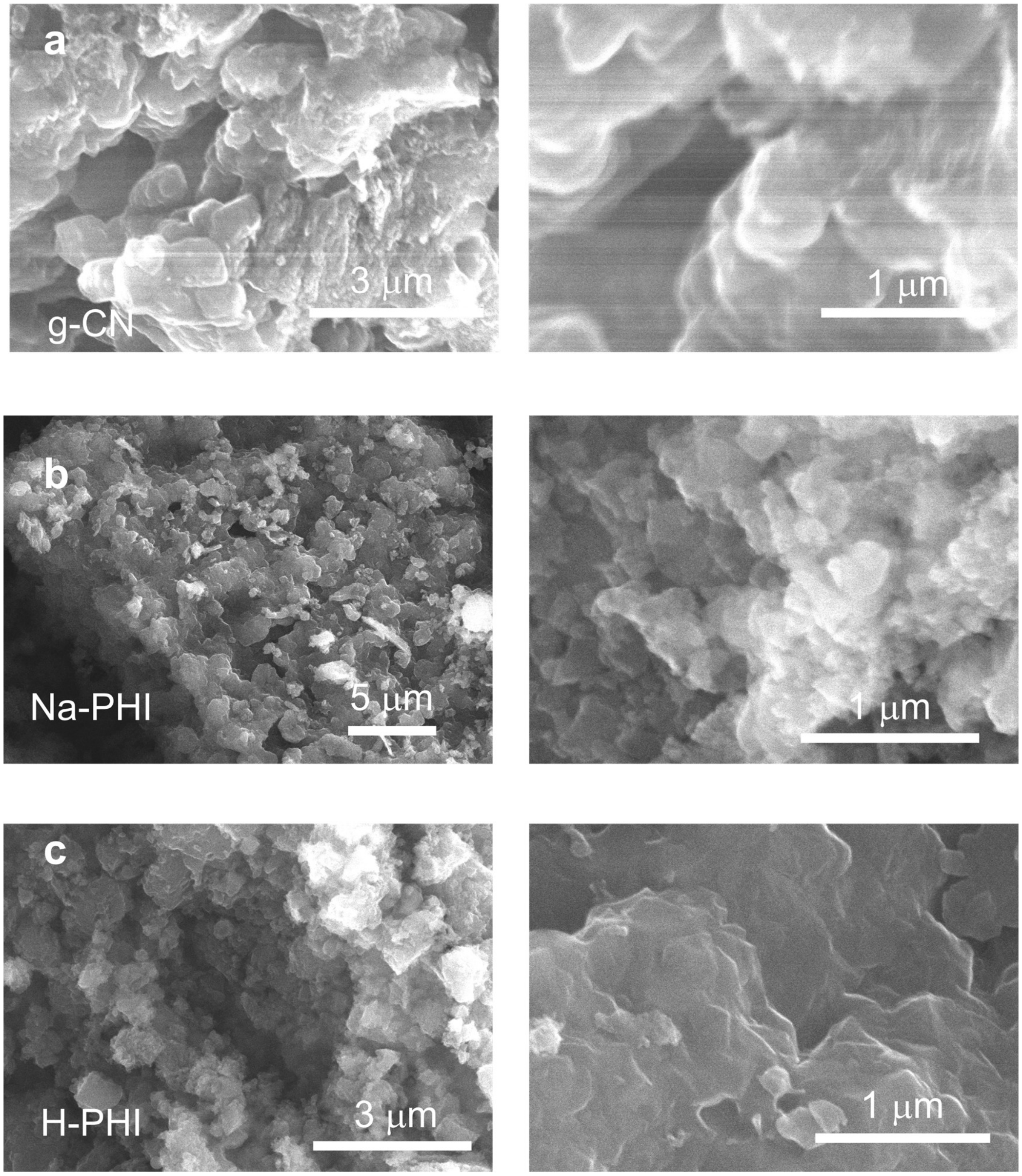

Supplementary Fig. 1. SEM images of the samples. a g-CN. **b** Na-PHI. **c** H-PHI.

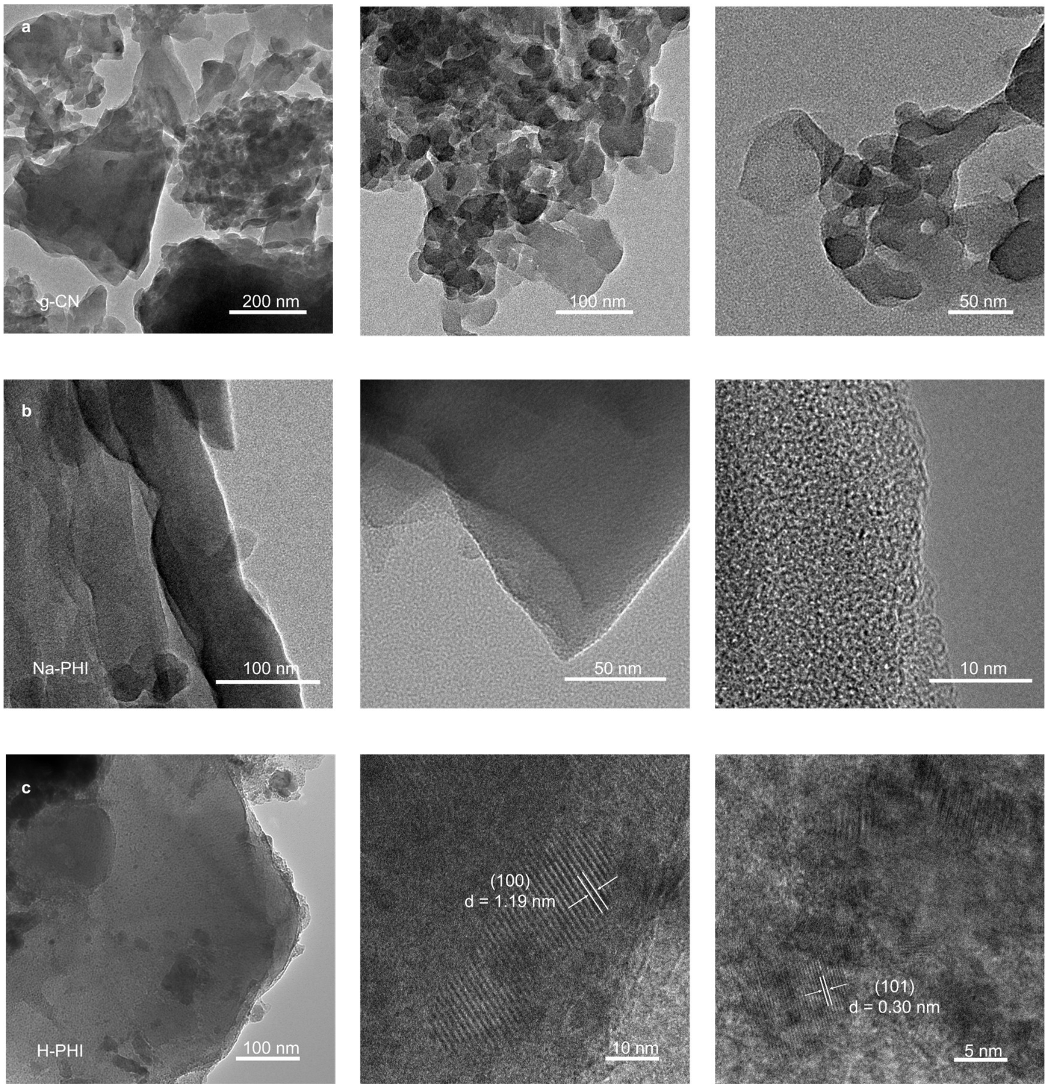

Supplementary Fig. 2. TEM images of the samples. a g-CN. **b** Na-PHI. **c** H-PHI.

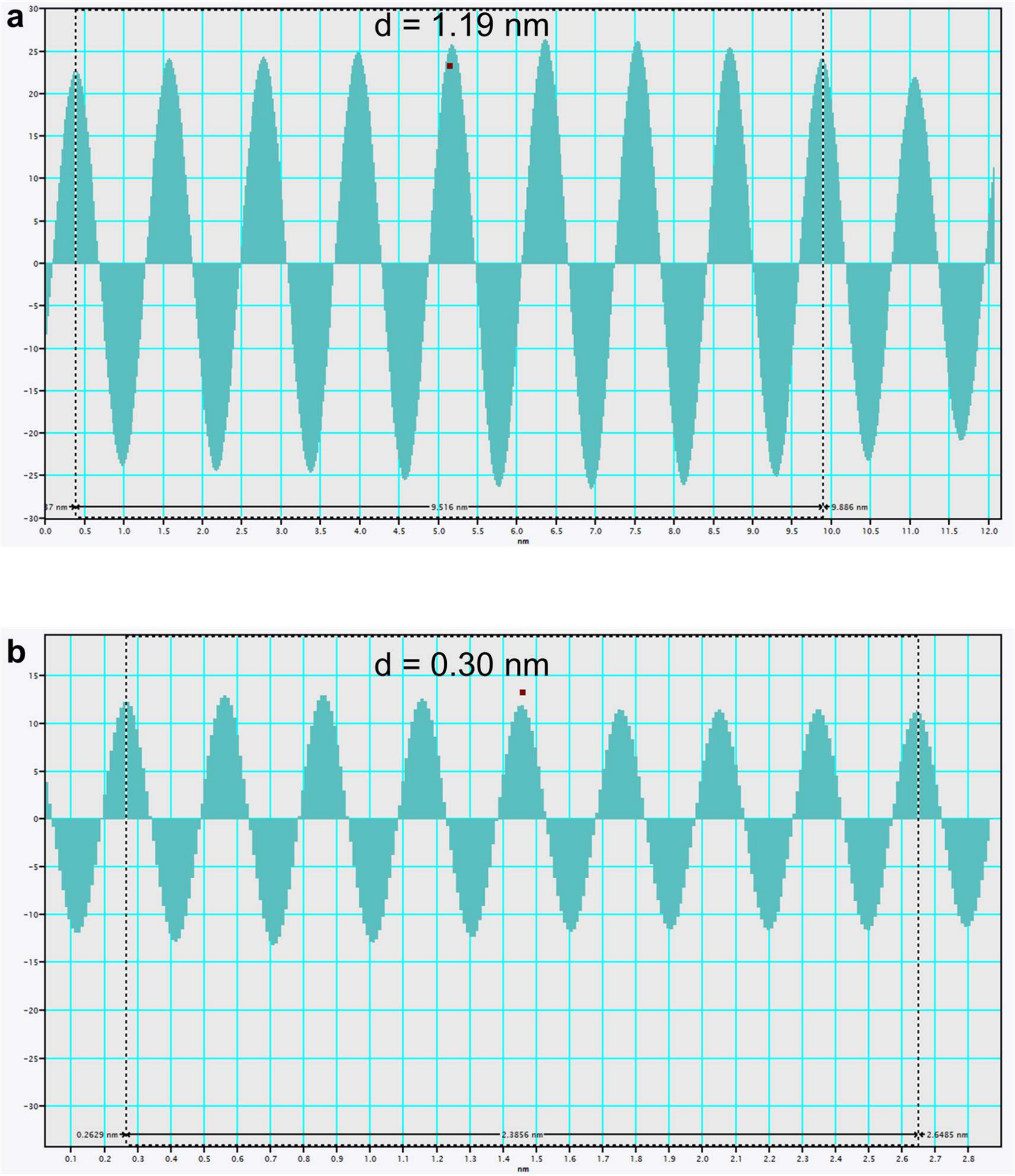

Supplementary Fig. 3. IFFT profile of HRTEM of H-PHI. a. IFFT profile corresponding to the (100) facet of H-PHI. **b.** IFFT profile corresponding to the (101) facet of H-PHI.

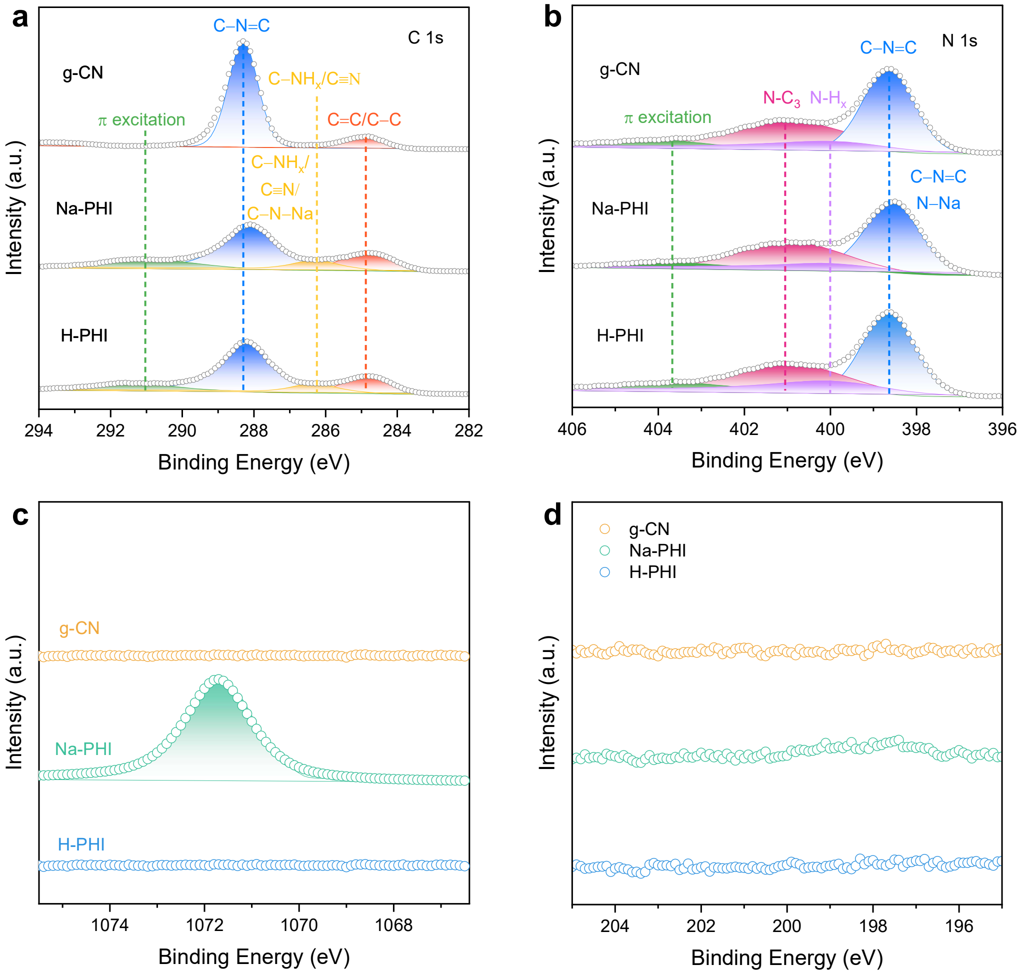

Supplementary Fig. 4. XPS spectra of the samples. **a** C 1s for the samples. **b** N 1s for the samples. **c** Na 1s for the samples. **d** Cl 2p for the samples.

In N 1s spectrum, the observed shift of the peak that is assigned to C−N=C moieties to higher binding energy may be explained by the conversion of the structure of carbon nitride from ionic to covalent triggered by acid treatment. Quenching of the negative charge on the PHI backbone upon protonation makes the structure more electron deficient. This observation agrees with that reported earlier.[44] Labeling the exact position of deprotonated imide moieties (N−Na) in N 1s spectra is challenging. Earlier it was reported that deprotonated imide fragments of PHI structure, in N 1s spectrum, are detected as a peak of weak intensity at 396.6 eV.[45]

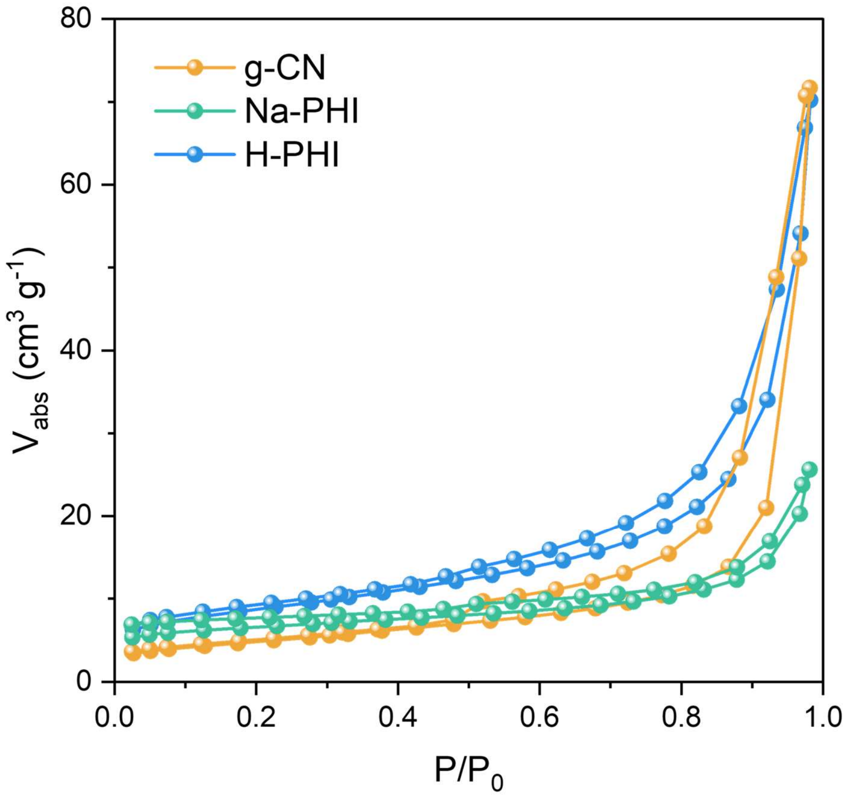

Supplementary Fig. 5. Nitrogen absorption-desorption isotherms of samples.

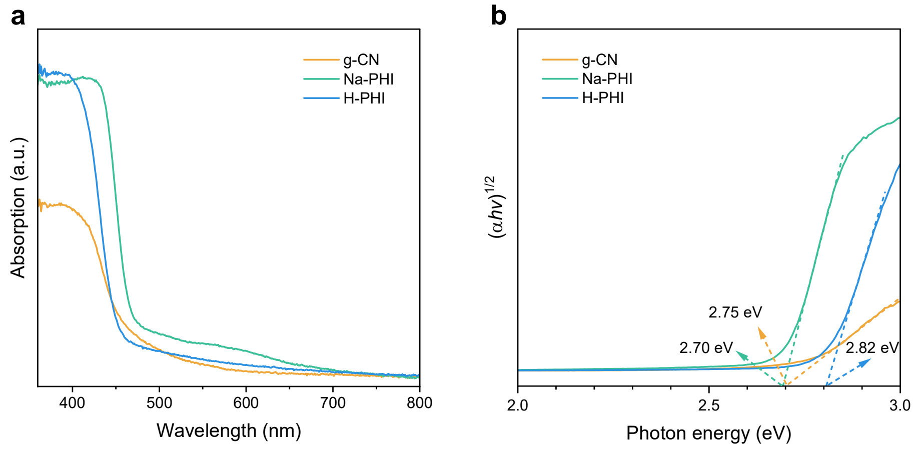

Supplementary Fig. 6. UV-vis measurement of the sample. a UV-vis DR spectra. **b** Band gaps of the samples.

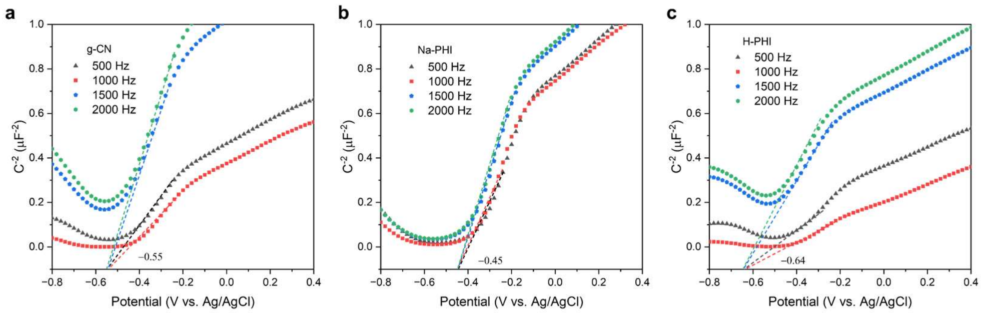

Supplementary Fig. 7. The Mott-Schottky curves of the samples. a g-CN. **b** Na-PHI. **c** H-PHI.

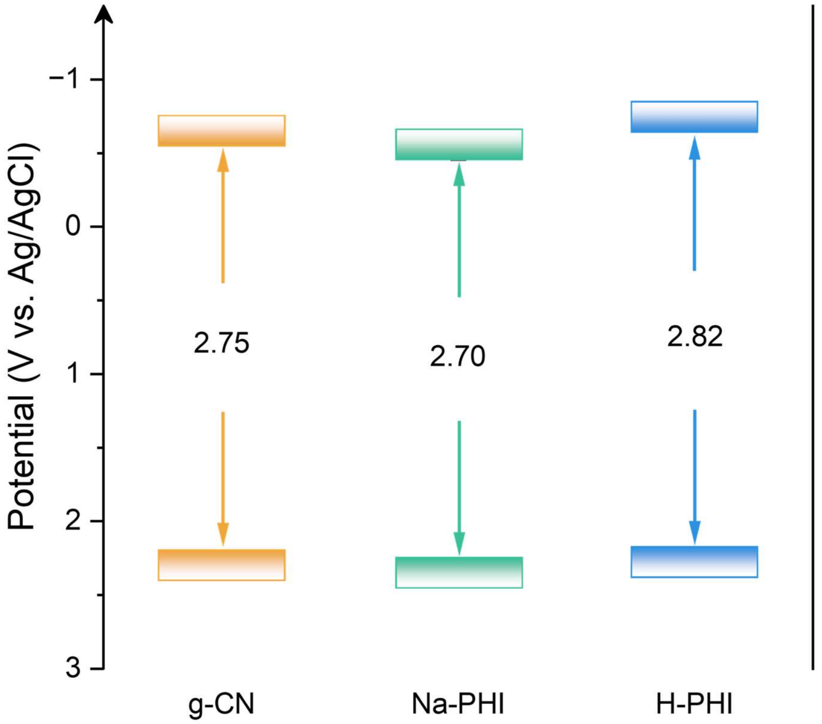

Supplementary Fig. 8. Schematic illustration of the band structures.

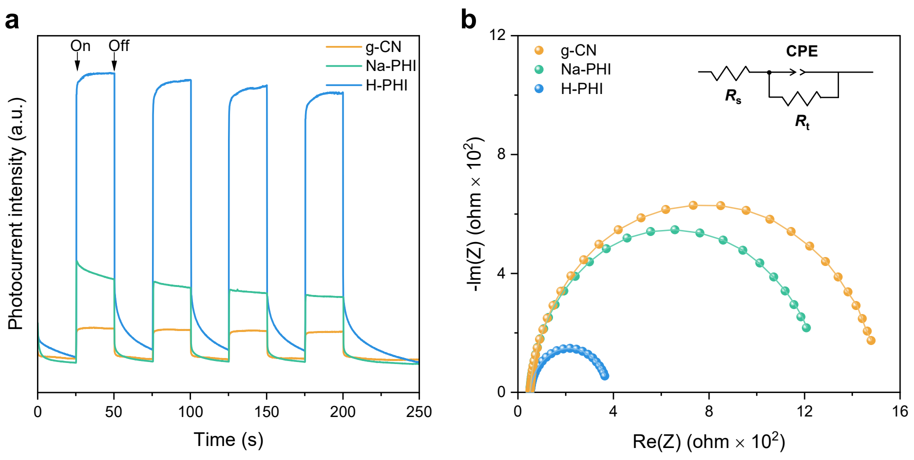

Supplementary Fig. 9. Photoelectrochemical measurements of the samples. a Photoelectrochemical response of the samples. **b** Nyquist plot of EIS with an equivalent circuit provided for data fitting. R_s, R_t, and CPE represent solution resistance, charge transfer resistance, and double-layer capacitance.

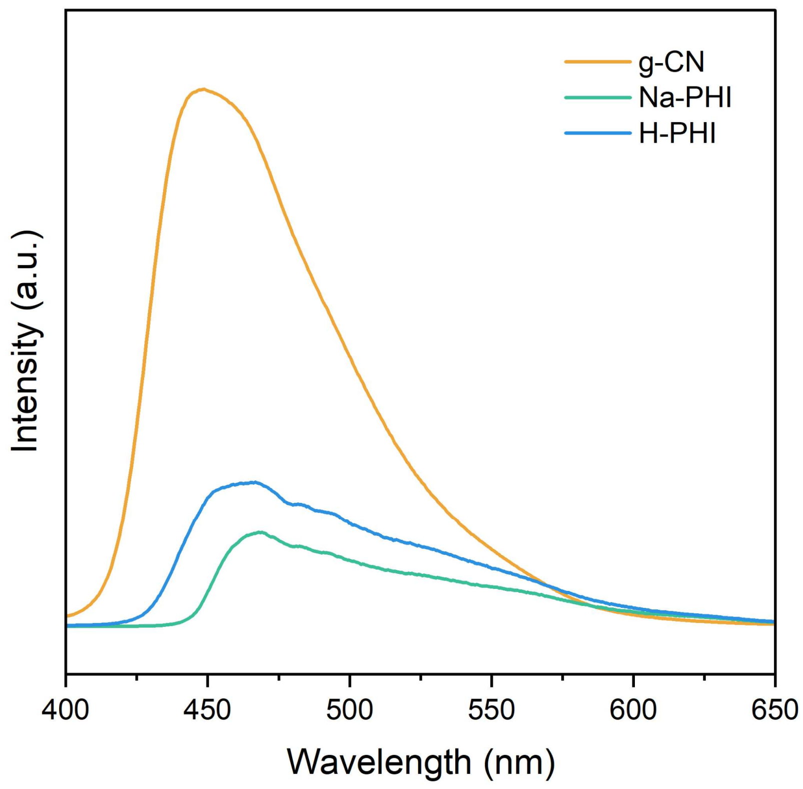

Supplementary Fig. 10. Photoluminescence spectra of the samples.

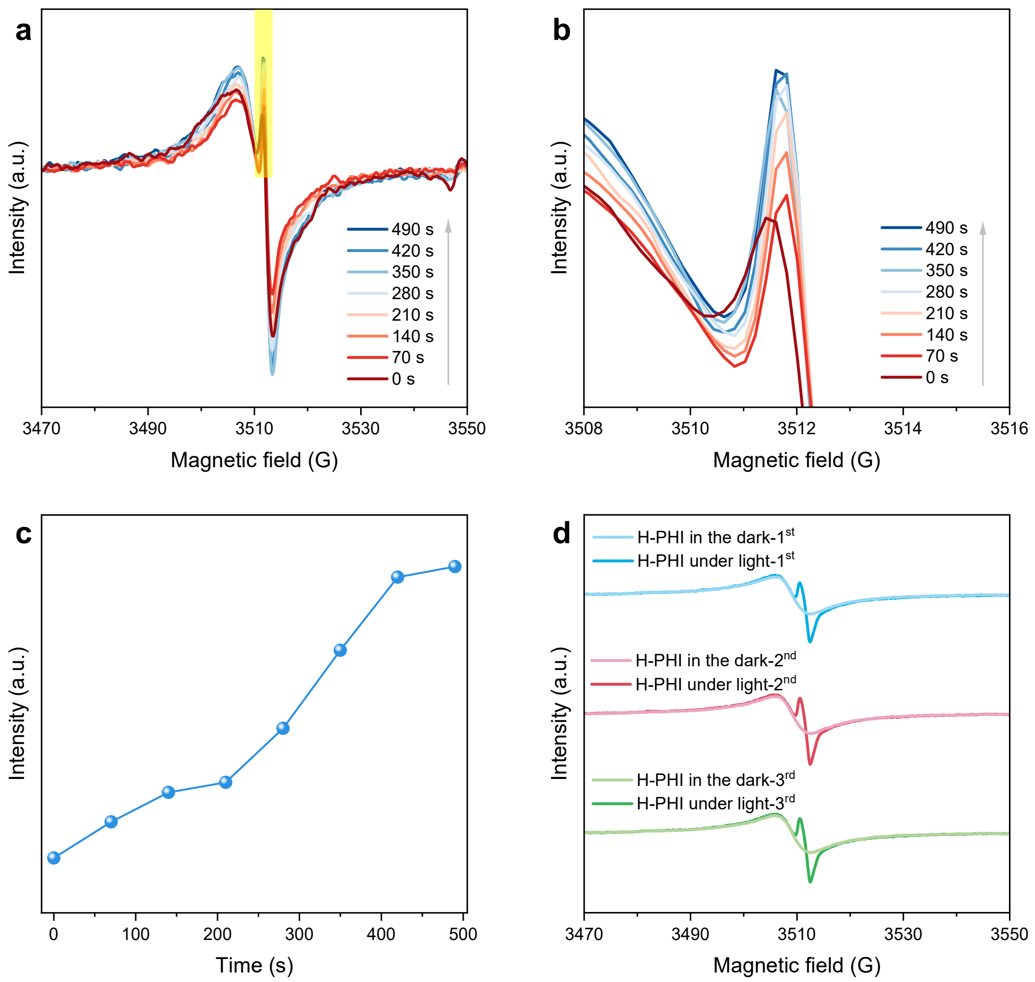

Supplementary Fig. 11. EPR measurements of H-PHI. a Evolution of EPR spectra of H-PHI over time acquired upon the sample continuous irradiation with light. Conditions: samples were recorded on CW EPR (X band) under central frequency (9.83 GHz) and attenuation (23 dB), 293 K. **b** Magnified region of the EPR spectra shown in panel “a” (highlighted with yellow background). **c** Plot of the EPR signal intensity observed at the magnetic field strength of 3511.7 G (highlighted region in panel “a” over time. **d** Formation of the additional signal at 3511.7 G triggered by H-PHI in irradiation at 410 nm and its immediate disappearance (in the dark), and reversibility of this phenomenon.

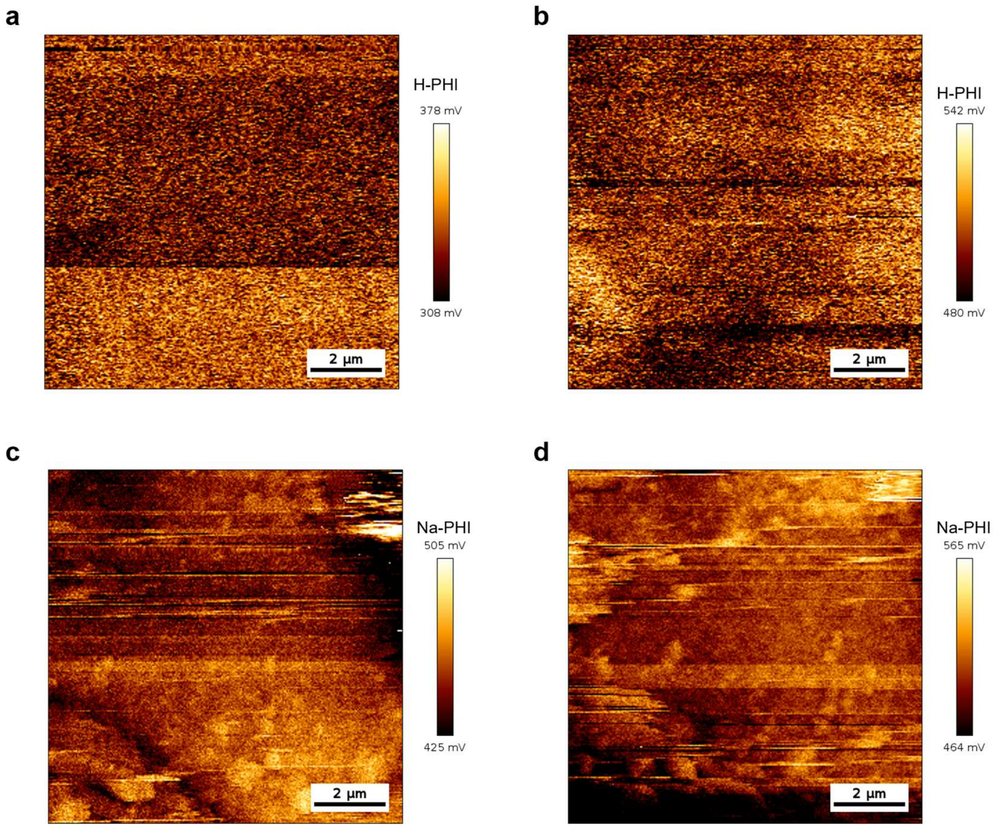

Supplementary Fig. 12. KPFM images of the samples. **a** H-PHI in the dark. **b** **H**-PHI under irradiation. **c** Na-PHI in the dark. **d** Na-PHI under irradiation. Light optical power (410 nm, 27.5±3 mW cm^{-2})**.**

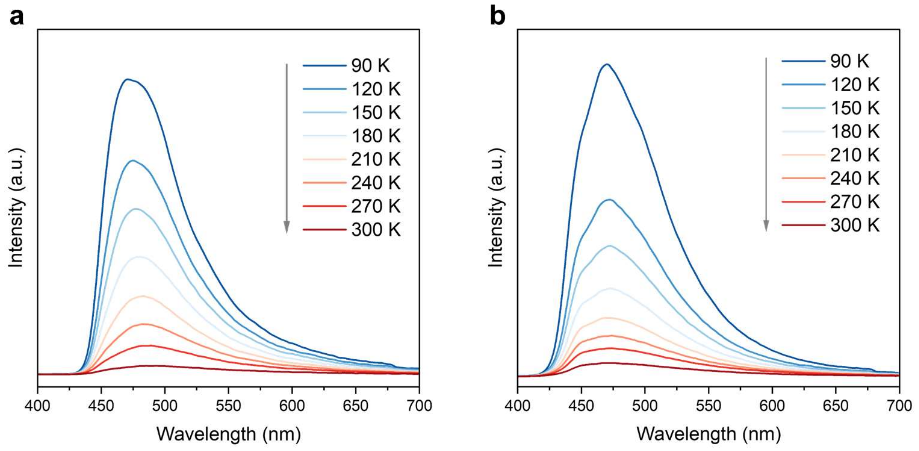

Supplementary Fig. 13. Temperature-dependent PL spectra. **a** Na-PHI. **b** H-PHI.

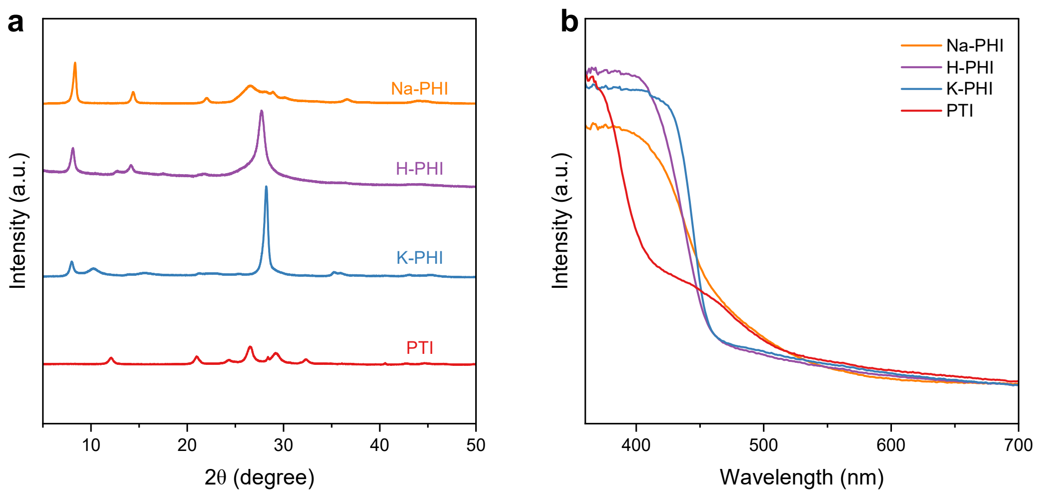

Supplementary Fig. 14. Characterizations of synthesized carbon nitride. a The XRD pattern of the samples. b UV-vis DR spectra of the samples.

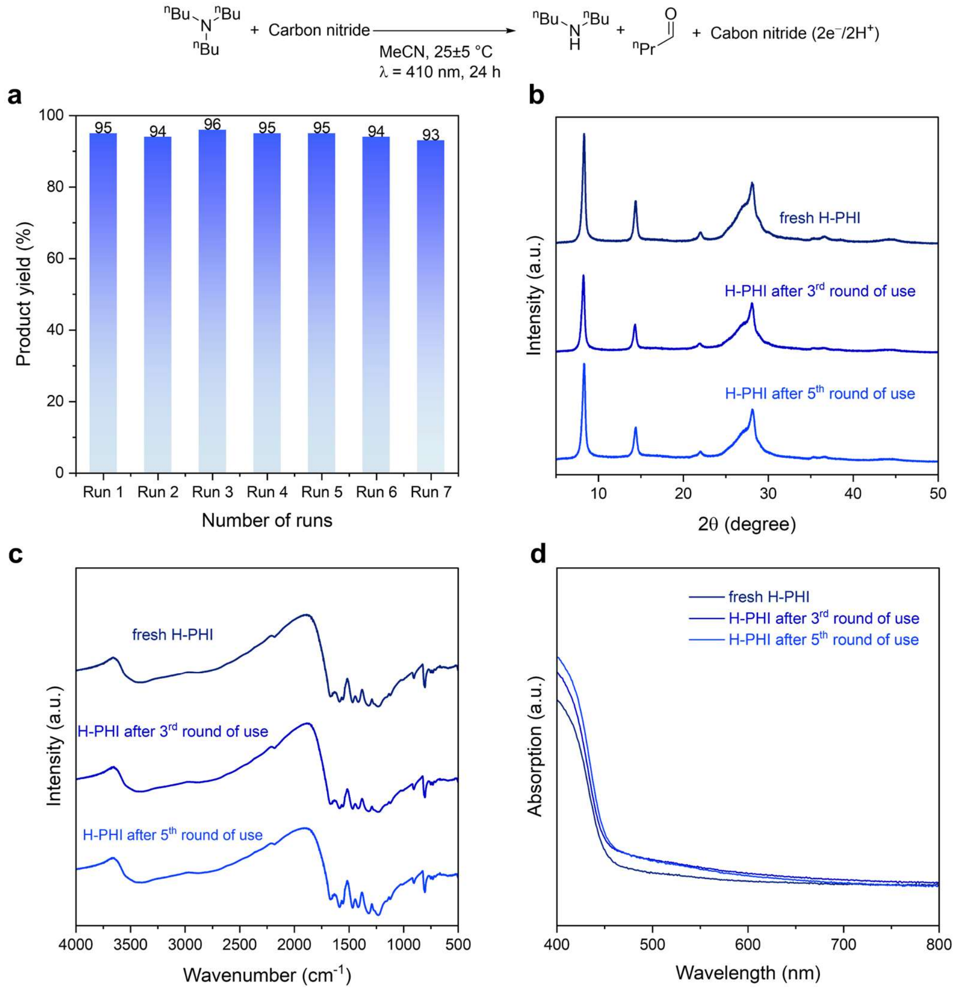

Supplementary Fig. 15. H-PHI recycling test. a H-PHI recycling result using (nBu)$_3$N as the substrate. Reaction condition: (nBu)$_3$N (0.05 mmol), H-PHI (320 mg), MeCN (2 mL), blue light (410 nm, 100 mW cm^{-2}), reaction time (24 h). **b** PXRD of fresh and recycled H-PHI. **c** FT-IR spectra of fresh and recycled H-PHI. **d** DRUV-vis spectra of fresh and recycled H-PHI.

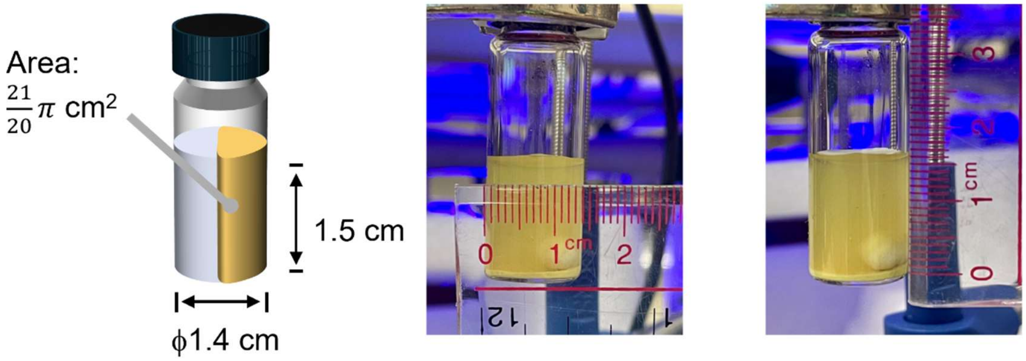

Supplementary Fig. 16. Irradiation area of the reaction mixture used in AQY calculations.

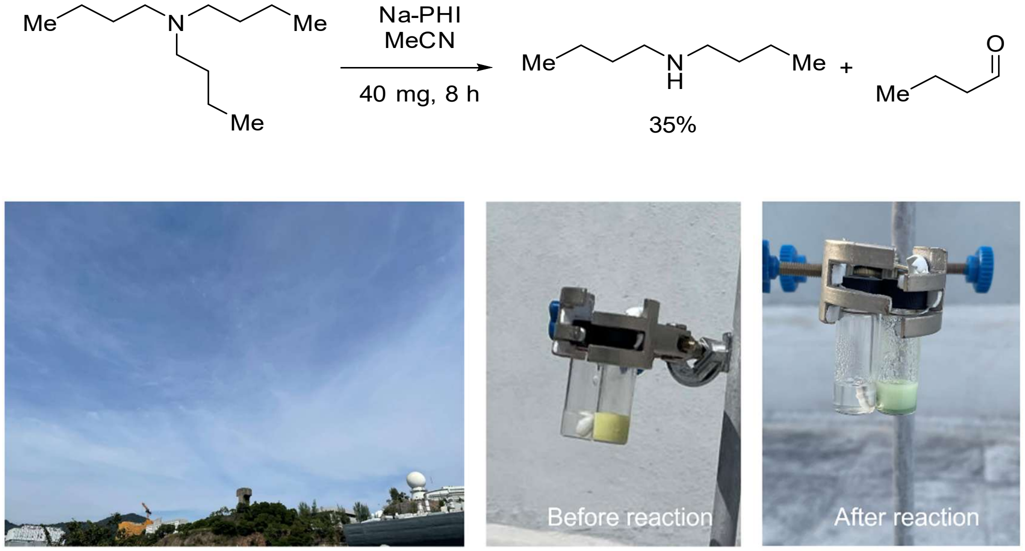

Supplementary Fig. 17. Outdoor reaction setup. Reaction condition: Na-PHI (40 mg), *n*Bu_3N (0.05 mmol), MeCN (2 mL).

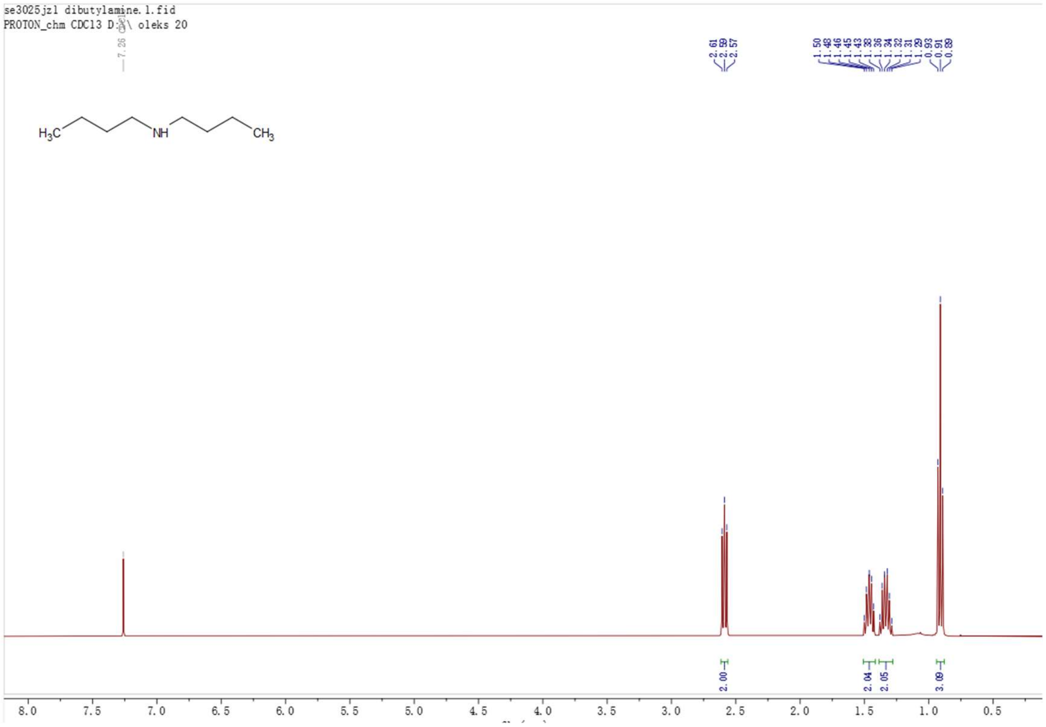

Supplementary Fig. 18. Dibutylamine ^{1}H NMR spectrum in $CDCl_3$.

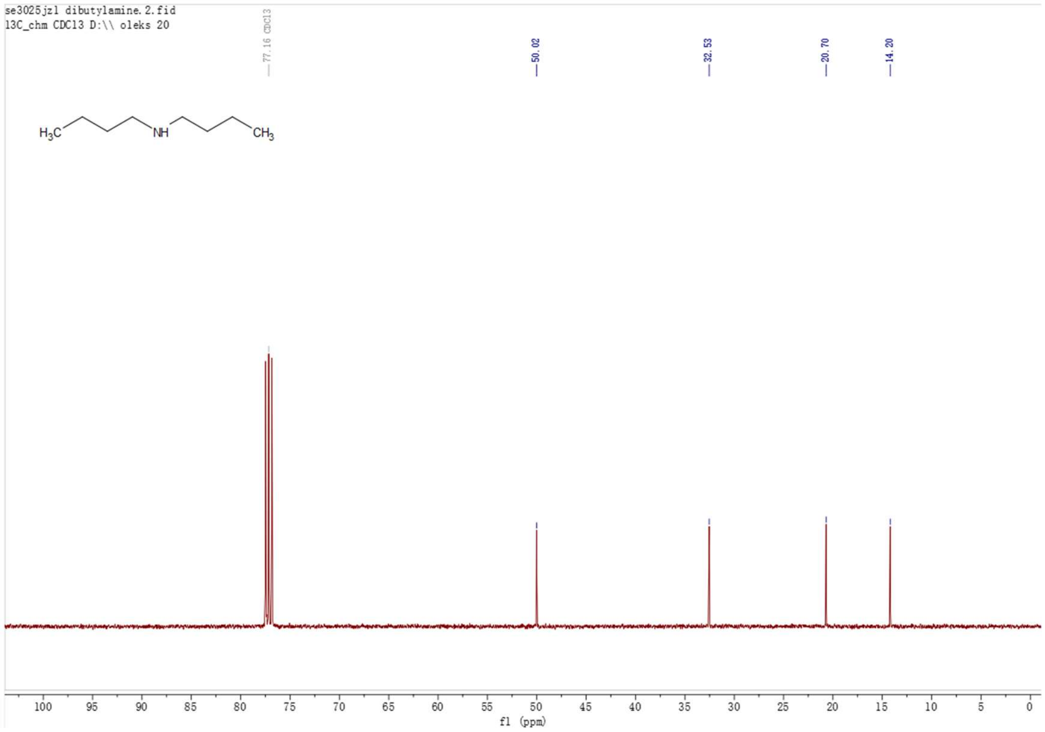

Supplementary Fig. 19. Dibutylamine 13C NMR spectrum in CDCl3.

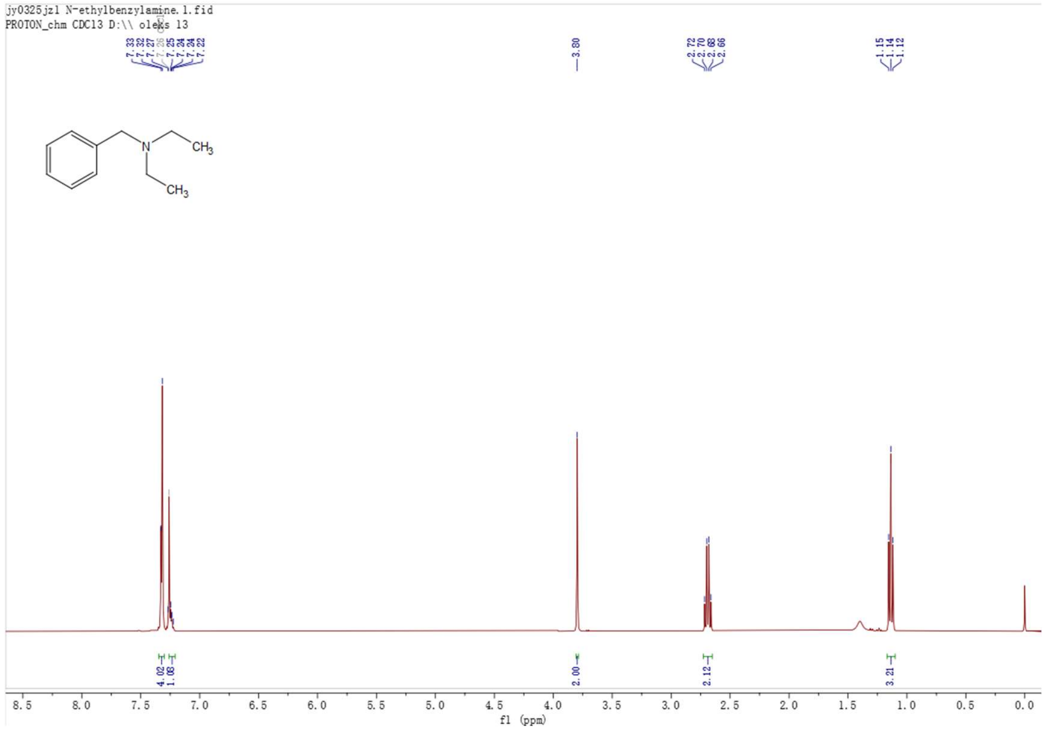

Supplementary Fig. 20. N,N-diethylbenzylamine 1H NMR spectrum in CDCl3.

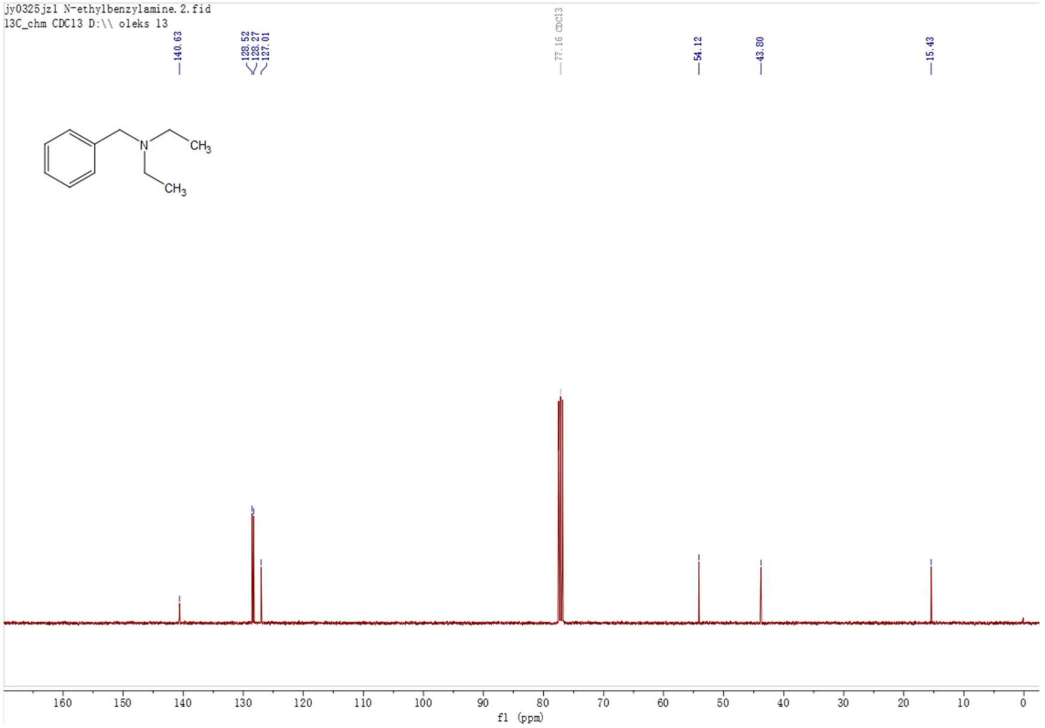

Supplementary Fig. 21. N,N-diethylbenzylamine 13C NMR spectrum in CDCl3.

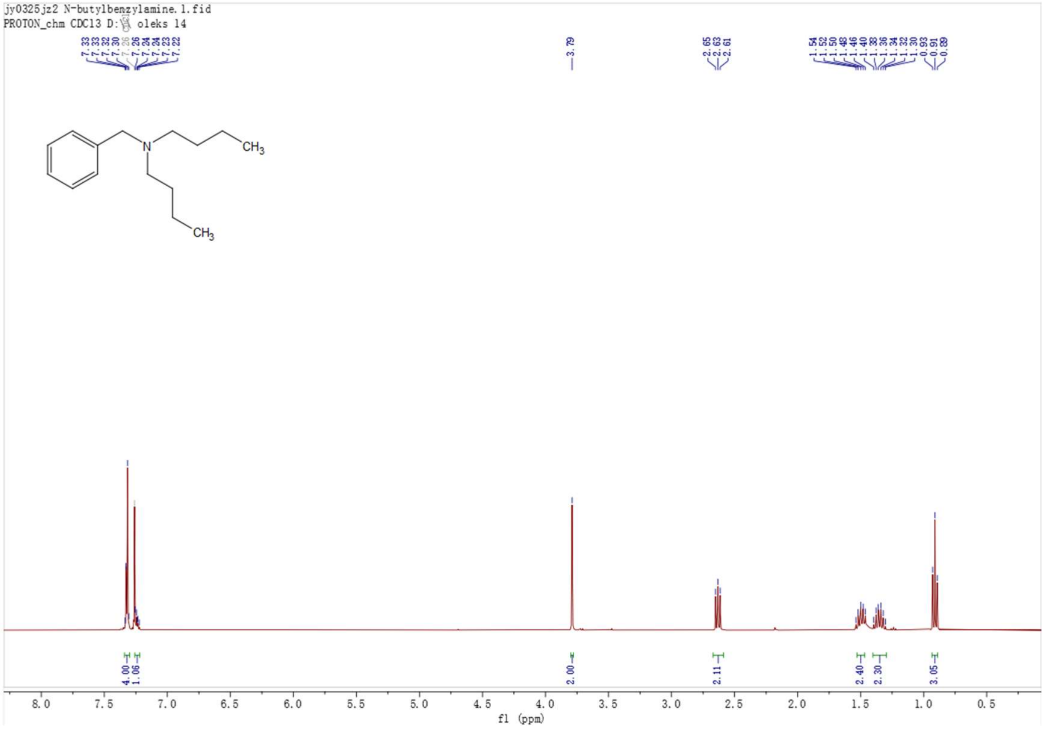

Supplementary Fig. 22. N,N-dibutylbenzylamine 1H NMR spectrum in CDCl3.

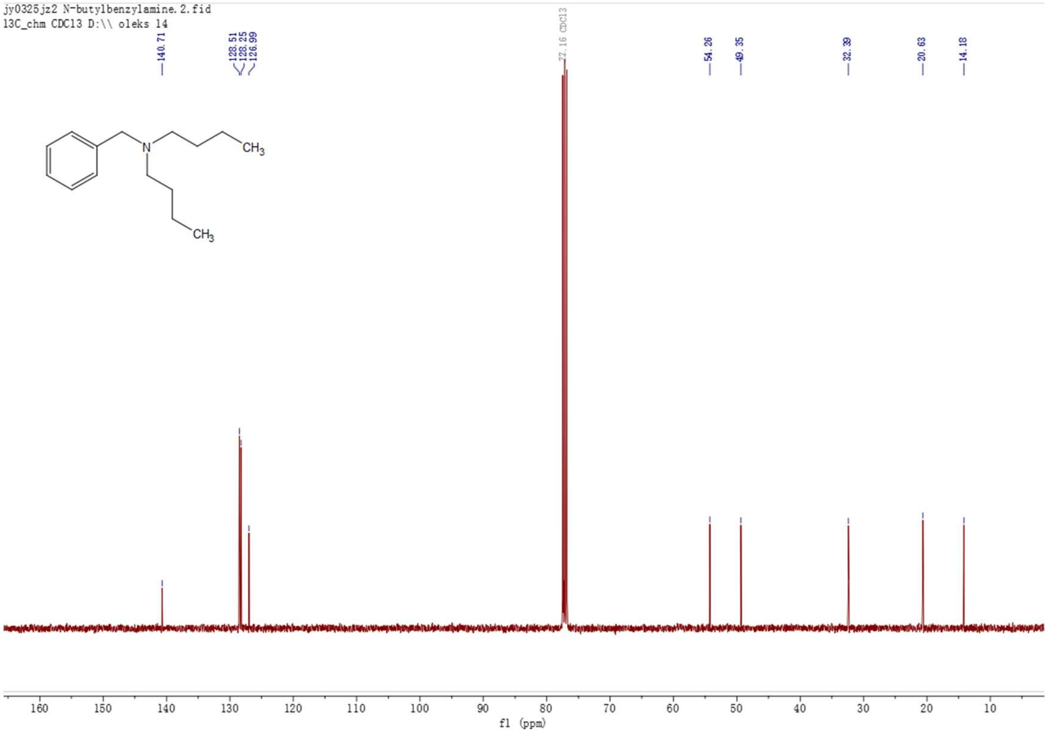

Supplementary Fig. 23. N,N-dibutylbenzylamine 13C NMR spectrum in CDCl3.

Supplementary Tables

Supplementary Table 1. Results from XRD measurement

Entry	Sample	Plane	Diffraction angle (°)	Interplanar spacing (Å)
1	g-CN	(210)	12.75	6.93
2	g-CN	(001)	27.68	3.22
3	Na-PHI	(100)	8.35	10.58
4	Na-PHI	(110)	14.39	6.15
5	Na-PHI	(001)	26.58	3.35
6	Na-PHI	(101)	28.98	3.08
7	H-PHI	(100)	8.33	10.61
8	H-PHI	(110)	14.37	6.15
	H-PHI	(001)	27.44	3.25
9	H-PHI	(101)	28.13	3.17

Supplementary Table 2. Physicochemical properties of the samples.

Sample	Specific surface area (m^2 g^{-1})	Pore volume (cm^3 g^{-1})	Average pore diameter (nm)
g-CN	17.0	0.03	6.8
Na-PHI	24.3	0.02	3.0
H-PHI	30.8	0.04	5.1

Supplementary Table 3. Result of KPFM study.[a]

Entry	Carbon nitride	Status (light on/off)	Minimum (mV)	Maximum (mV)	Average (mV)
1	Na-PHI	Light off	424.8	505.0	448.5
2	Na-PHI	Light on	464.5	565.4	494.3
3	H-PHI	Light off	308.0	377.5	328.5
4	H-PHI	Light on	480.2	542.2	498.5

[a] Light intensity (410 nm, 27.5±3 mW cm^{-2}).

Supplementary Table 4. Selected conditions for triethylamine dealkylation screening.

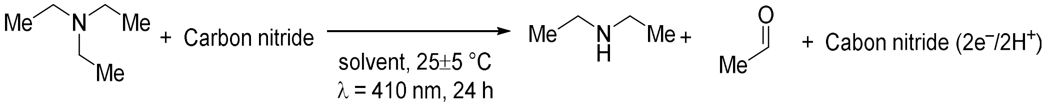

Entry	TEA (mmol)	Carbon nitride	Solvent	Yield (%) [a]
1	0.2	Na-PHI	MeOH	9
		H-PHI		5
2	0.2	Na-PHI	$CHCl_3$	12
		H-PHI		12
3	0.2	Na-PHI	CH_3NO_2	20
		H-PHI		22
4	0.2	Na-PHI	DMSO	8
		H-PHI		9
5	0.2	Na-PHI	MeCN	8
		H-PHI		9
6	0.2	Na-PHI	THF	Traces
		H-PHI		
7	0.2	Na-PHI	MeCN:H_2O = 1:1	8
		H-PHI		9
8[b]	0.05	PTI	MeCN	12
9	0.05	Na-PHI	MeOH	12
		H-PHI		10

Reaction condition: Triethylamine (TEA, specific amount), semiconductor (20 mg), solvent (2 mL), blue light (410 nm, 100 mW cm^{-2}), reaction time (24 h).

[a] Yields were determined from ^{1}H NMR spectra using 1,3,5-trimethoxybenzene as internal standard.

[b] UV light (365 nm, 108 mW cm^{-2}).

Supplementary Table 5. Screening of the amount of carbon nitride.

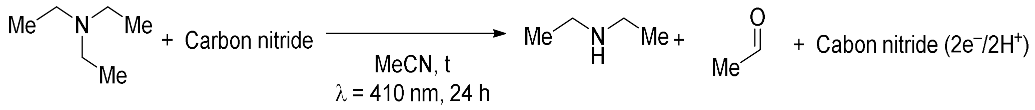

Entry	Carbon nitride	Specific surface area (m^2 g^{-1})	Mass (mg)	N_{total} (μmol g^{-1})	N_{hept} (μmol)	t (°C)	Conversion (%)[a]	Yield (%)[a]	Electron storage (μmol)
1	Na-PHI	24.3	1	4914	4.9	25±5	16	15.6	15.6
2	Na-PHI	24.3	2	4914	9.8	25±5	20.5	16.6	16.6
3	Na-PHI	24.3	5	4914	24.6	25±5	21.8	21.5	21.5
4	Na-PHI	24.3	10	4914	49.1	25±5	27	22.5	22.5
5	Na-PHI	24.3	20	4914	98.3	25±5	31	24.9	24.9
6	Na-PHI	24.3	40	4914	196.6	25±5	32.9	32.2	32.2
7	Na-PHI	24.3	80	4914	393.1	25±5	67.4	56	56
8	Na-PHI	24.3	160	4914	786.2	25±5	73.1	60.6	60.6
9	Na-PHI	24.3	320	4914	1572.5	25±5	87.4	69.6	69.8
10	Na-PHI	24.3	160	4914	786.2	80	67	50	50
11	Na-PHI	24.3	320	4914	1572.5	80	85	49	49
12	H-PHI	30.8	1	5195	5.2	25±5	24.4	17.6	17.6
13	H-PHI	30.8	2	5195	10.4	25±5	34.2	20.5	20.5

14	H-PHI	30.8	5	5195	26.0	25±5	36.2	22.5	22.5
15	H-PHI	30.8	10	5195	52.0	25±5	37.5	23.5	23.5
16	H-PHI	30.8	20	5195	103.9	25±5	39.3	32.1	32.1
17	H-PHI	30.8	40	5195	207.8	25±5	41.4	33.2	33.2
18	H-PHI	30.8	80	5195	415.6	25±5	54	53.4	53.4
19	H-PHI	30.8	160	5195	831.2	25±5	100	75	75
20	H-PHI	30.8	320	5195	1662.34	25±5	100	99	99
21	H-PHI	30.8	160	5195	831.2	80	100	52.1	52.1
22	H-PHI	30.8	320	5195	1662.34	80	100	64.5	64.5

Reaction condition: Triethylamine (TEA, specific amount), semiconductor (20 mg), solvent (2 mL), blue light (410 nm, 100 mW cm^{-2}), reaction time (24 h), N_2.

[a] Yields and conversions were determined from ^1H NMR spectra using 1,3,5-trimethoxybenzene as internal standard. For Na-PHI, M_w = 407 g mol^{-1}; H-PHI, M_w = 385 g mol^{-1}.

Supplementary Table 6. Photocharging rate of PHIs in the TEA dealkylation in every four hours.

Stage	Period (h)	Carbon nitride	ΔN_e (μmol)	Photocharging rate (μmol min^{-1})
1	0 - 4	Na-PHI	7.2	0.030
		H-PHI	11.2	0.046
2	4 - 8	Na-PHI	4.6	0.019
		H-PHI	5.6	0.024
3	8 - 12	Na-PHI	3.9	0.016
		H-PHI	4.6	0.019
4	12 - 16	Na-PHI	1.9	0.008
		H-PHI	2.6	0.011
5	16 - 20	Na-PHI	1.4	0.006
		H-PHI	2.7	0.011
6	20 - 24	Na-PHI	3.9	0.016
		H-PHI	5.2	0.022

Reaction condition: Triethylamine (TEA, 0.05 mmol), Carbon nitride (20 mg), MeCN (2 mL), blue light (410 nm, 100 mW cm^{-2}), reaction time (specific time), N_2.

Supplementary Table 7. Photochemical dealkylation of (nBu$_3$)N driven photons of different wavelengths.

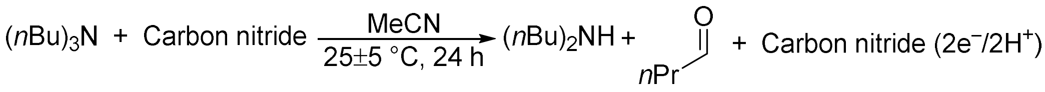

Carbon nitride	Photon wavelength (nm)	Mass (mg)	Reaction time (h)	Product yield (%)[a]
H-PHI	410[b]	20	5	16
H-PHI	Full spectrum[c]	20	5	27
H-PHI	410[b]	320	5	63
H-PHI	Full spectrum[c]	320	5	75

Reaction condition: (nBu)$_3$N (0.05 mmol), H-PHI (specific amount), MeCN (2 mL), reaction time (5 h), N_2.

[a] Yields were determined from ^{1}H NMR spectra using 1,3,5-trimethoxybenzene as internal standard.

[b] Blue LED (410 nm, 100 mW cm^{-2}).

[c] Xe lamp (300 W electric power, optical power approx. 100 mW cm^{-2})

References

[1] Z. Chen, A. Savateev, S. Pronkin, V. Papaefthimiou, C. Wolff, M. G. Willinger, E. Willinger, D. Neher, M. Antonietti, D. Dontsova, “The Easier the Better” Preparation of Efficient Photocatalysts—Metastable Poly(heptazine imide) Salts, *Adv. Mater.,* **2017**, *29*.

[2] A. Savateev, S. Pronkin, M. G. Willinger, M. Antonietti, D. Dontsova, Towards Organic Zeolites and Inclusion Catalysts: Heptazine Imide Salts Can Exchange Metal Cations in the Solid State, *Chem. -Asian J.,* **2017**, *12*, 1517-1522.

[3] V. Shvalagin, N. Tarakina, B. Badamdorj, I.-M. Lahrsen, E. Bargiacchi, A. Bardow, Z. Deng, W. Wang, D. L. Phillips, Z. Guo, G. Zhang, J. Tang, O. Savateev, Simultaneous Photocatalytic Production of H2 and Acetal from Ethanol with Quantum Efficiency over 73% by Protonated Poly(heptazine imide) under Visible Light, *ACS Catal.,* **2024**, *14*, 14836-14854.

[4] C. Wang, N. Shi, Y. Zhou, Y. Lu, J. Zhuang, H. Liao, C. Ye, H. Lei, X. Lin, J. Zheng, T. X. Liu, Z. Yuan, Large-Sized Poly (Triazine Imide) Crystals with Minimized Defects for High-Efficiency Overall Water Splitting, *Adv. Sci.,* **2025**, *12*, e10084.

[5] L. Xu, K. S. Yeung, L. Li, X. Nan, O. Savateev, Z. Hu, J. C. Yu, Production of H2O2 via Energy Transfer Photocatalysis by Coupling with Furfuryl Alcohol Conversion over an Amide-Functionalized Heptazine Framework, *Angew. Chem. Int. Ed.*, e202504635.

[6] O. Savateev, K. Nolkemper, T. D. Kuhne, V. Shvalagin, Y. Markushyna, M. Antonietti, Extent of carbon nitride photocharging controls energetics of hydrogen transfer in photochemical cascade processes, *Nat. Commun.,* **2023**, *14*, 7684.

[7] Y. Jin, D. Zheng, Z. Fang, Z. Pan, S. Wang, Y. Hou, O. Savateev, Y. Zhang, G. Zhang, Salt-melt synthesis of poly(heptazine imide) in binary alkali metal bromides for enhanced visible-light photocatalytic hydrogen production, *Interdiscip. Mater.,* **2024**, *3*, 389-399.

[8] P. Zheng, R. Zubatyuk, W. Wu, O. Isayev, P. O. Dral, Artificial intelligence-enhanced quantum chemical method with broad applicability, *Nat. Commun.,* **2021**, *12*, 7022.

[9] Y. P. O. Chen, P. O. Dral, AIQM2: Organic Reaction Simulations Beyond DFT, *Chem. Sci.,* **2025**.

[10] P. O. Dral, F. Ge, Y.-F. Hou, P. Zheng, Y. Chen, M. Barbatti, O. Isayev, C. Wang, B.-X. Xue, M. Pinheiro, Y. Su, Y. Dai, Y. Chen, L. Zhang, S. Zhang, A. Ullah, Q. Zhang, Y. Ou, MLatom 3: Platform for machine learning-enhanced computational chemistry simulations and workflows, *J. Chem. Theory Comput.,* **2023**, *20*, 1193–1213.

[11] Gaussian 16 Rev. A.01, Wallingford, CT, 2016,

[12] J. Hu, H. Nawaz, Y. Rui, L. Chi, A. Ullah, P. O. Dral, Aitomia: Your Intelligent Assistant for AI-Driven Atomistic and Quantum Chemical Simulations, *ChemRxiv,* **2025**.

[13] Semiempirical extended tight-binding program package xtb. https://github.com/grimme-lab/xtb

(accessed on Nov. 19, 2022).

[14] C. Bannwarth, S. Ehlert, S. Grimme, GFN2-xTB-An Accurate and Broadly Parametrized Self-Consistent Tight-Binding Quantum Chemical Method with Multipole Electrostatics and Density-Dependent Dispersion Contributions, *J. Chem. Theory Comput.,* **2019**, *15*, 1652–1671.

[15] P. O. Dral, X. Wu, W. Thiel, Semiempirical Quantum-Chemical Methods with Orthogonalization and Dispersion Corrections, *J. Chem. Theory Comput.,* **2019**, *15*, 1743-1760.

[16] MNDO2020: a semiempirical quantum chemistry program, Max-Planck-Institut für Kohlenforschung, Mülheim an der Ruhr, 2020, https://mndo.kofo.mpg.de, January 11, 2023

[17] E. Caldeweyher, C. Bannwarth, S. Grimme, Extension of the D3 dispersion coefficient model, *J. Chem. Phys.,* **2017**, *147*, 034112.

[18] DFT-D4, Version 2.5.0, Mulliken Center for Theoretical Chemistry, University of Bonn, 2020,

[19] X. Gao, F. A.-O. Ramezanghorbani, O. A.-O. Isayev, J. S. Smith, A. A.-O. Roitberg, TorchANI: A Free and Open Source PyTorch-Based Deep Learning Implementation of the ANI Neural Network Potentials, *J. Chem. Inf. Model.*, *60*, 3408-3415.

[20] J. D. Chai, M. Head-Gordon, Long-range corrected hybrid density functionals with damped atom-atom dispersion corrections, *Phys Chem Chem Phys,* **2008**, *10*, 6615-6620.

[21] A. Schäfer, H. Horn, R. Ahlrichs, Fully optimized contracted Gaussian basis sets for atoms Li to Kr, *J. Chem. Phys.,* **1992**, *97*, 2571-2577.

[22] A. Schäfer, C. Huber, R. Ahlrichs, Fully optimized contracted Gaussian basis sets of triple zeta valence quality for atoms Li to Kr, *J. Chem. Phys.,* **1994**, *100*, 5829-5835.

[23] F. Weigend, R. Ahlrichs, Balanced basis sets of split valence, triple zeta valence and quadruple zeta valence quality for H to Rn: Design and assessment of accuracy, *Phys. Chem. Chem. Phys.,* **2005**, *7*, 3297-3305.

[24] F. Weigend, Accurate Coulomb-fitting basis sets for H to Rn, *Phys. Chem. Chem. Phys.,* **2006**, *8*, 1057-1065.

[25] A. D. Becke, Density‐functional thermochemistry. III. The role of exact exchange, *J. Chem. Phys.,* **1993**, *98*, 5648-5652.

[26] P. J. Stephens, F. J. Devlin, C. F. Chabalowski, M. J. Frisch, Ab Initio Calculation of Vibrational Absorption and Circular Dichroism Spectra Using Density Functional Force Fields, *J. Phys. Chem.,* **1994**, *98*, 11623-11627.

[27] P. C. Hariharan, J. A. Pople, The influence of polarization functions on molecular orbital hydrogenation energies, *Theor. Chim. Acta,* **1973**, *28*, 213-222.

[28] W. J. Hehre, R. Ditchfield, J. A. Pople, Self—Consistent Molecular Orbital Methods. XII. Further

Extensions of Gaussian—Type Basis Sets for Use in Molecular Orbital Studies of Organic Molecules, *J. Chem. Phys.,* **1972**, *56*, 2257-2261.

[29] V. Barone, M. Cossi, Quantum calculation of molecular energies and energy gradients in solution by a conductor solvent model, *J. Phys. Chem. A,* **1998**, *102*, 1995-2001.

[30] M. Cossi, N. Rega, G. Scalmani, V. Barone, Energies, structures, and electronic properties of molecules in solution with the C-PCM solvation model, *J. Comput. Chem.,* **2003**, *24*, 669-681.

[31] R. Bauernschmitt, R. Ahlrichs, Treatment of electronic excitations within the adiabatic approximation of time dependent density functional theory, *Chem. Phys. Lett.,* **1996**, *256*, 454-464.

[32] M. E. Casida, C. Jamorski, K. C. Casida, D. R. Salahub, Molecular excitation energies to high-lying bound states from time-dependent density-functional response theory: Characterization and correction of the time-dependent local density approximation ionization threshold, *J. Chem. Phys.,* **1998**, *108*, 4439-4449.

[33] R. E. Stratmann, G. E. Scuseria, M. J. Frisch, An efficient implementation of time-dependent density-functional theory for the calculation of excitation energies of large molecules, *J. Chem. Phys.,* **1998**, *109*, 8218-8224.

[34] C. Van Caillie, R. D. Amos, Geometric derivatives of excitation energies using SCF and DFT, *Chem. Phys. Lett.,* **1999**, *308*, 249-255.

[35] C. Van Caillie, R. D. Amos, Geometric derivatives of density functional theory excitation energies using gradient-corrected functionals, *Chem. Phys. Lett.,* **2000**, *317*, 159-164.

[36] F. Furche, R. Ahlrichs, Adiabatic time-dependent density functional methods for excited state properties, *J. Chem. Phys.,* **2002**, *117*, 7433-7447.

[37] G. Scalmani, M. J. Frisch, B. Mennucci, J. Tomasi, R. Cammi, V. Barone, Geometries and properties of excited states in the gas phase and in solution: Theory and application of a time-dependent density functional theory polarizable continuum model, *J. Chem. Phys.,* **2006**, *124*.

[38] K. Burke, J. Werschnik, E. K. U. Gross, Time-dependent density functional theory: Past, present, and future, *J. Chem. Phys.,* **2005**, *123*.

[39] R. S. Mulliken, Electronic Population Analysis on LCAO–MO Molecular Wave Functions. I, *J. Chem. Phys.,* **1955**, *23*, 1833-1840.

[40] J. Ehrmaier, E. J. Rabe, S. R. Pristash, K. L. Corp, C. W. Schlenker, A. L. Sobolewski, W. Domcke, Singlet–Triplet Inversion in Heptazine and in Polymeric Carbon Nitrides, *The Journal of Physical Chemistry A,* **2019**, *123*, 8099-8108.

[41] O. Savateev, J. Zhuang, A Guide to Chemical Reactions Design in Carbon Nitride Photocatalysis, *ChemPhotoChem,* **2024**, *8*, e202300306.

[42] P. Zheng, R. Zubatyuk, W. Wu, O. Isayev, P. O. Dral, Artificial Intelligence-Enhanced Quantum Chemical Method with Broad Applicability, *Nat. Commun.,* **2021**, *12*, 7022.

[43] P. Zheng, W. Yang, W. Wu, O. Isayev, P. O. Dral, Toward Chemical Accuracy in Predicting Enthalpies of Formation with General-Purpose Data-Driven Methods, *J. Phys. Chem. Lett.,* **2022**, *13*, 3479–3491.

[44] Q. Deng, H. Li, W. Hu, W. Hou, Stability and Crystallinity of Sodium Poly(Heptazine Imide) in Photocatalysis, *Angew. Chem. Int. Ed.,* **2023**, *62*, e202314213.

[45] D. Dontsova, S. Pronkin, M. Wehle, Z. Chen, C. Fettkenhauer, G. Clavel, M. Antonietti, Triazoles: A New Class of Precursors for the Synthesis of Negatively Charged Carbon Nitride Derivatives, *Chem. Mater,* **2015**, *27*, 5170-5179.
